# Application of enhanced recovery after surgery in partial nephrectomy for renal tumors: A systematic review and meta-analysis

**DOI:** 10.3389/fonc.2023.1049294

**Published:** 2023-02-09

**Authors:** Wu Wangjian, Lu Tianyi, Ma Xiaoqian, Zhang Di, Zhou Chuan, Wang Chao, Da Zijian, Jin Tongtong, Zhou Fenghai

**Affiliations:** ^1^ The First Clinical Medical College of Lanzhou University, Lanzhou, China; ^2^ The First Clinical Medical College of Gansu University of Chinese Medicine (Gansu Provincial Hospital), Lanzhou, China; ^3^ Department of Pediatrics, The First Affiliated Hospital of Bengbu Medical College, Bengbu, China; ^4^ Department of Urology, Gansu Provincial Hospital, Lanzhou, China

**Keywords:** renal tumors, partial nephrectomy, meta-analysis, enhanced recovery after surgery, systematic review

## Abstract

**Objectives:**

In recent years, enhanced recovery after surgery (ERAS) has been widely used in the field of urology, especially in radical cystectomy and radical prostatectomy, and has demonstrated its advantages. Although studies on the application of ERAS in partial nephrectomy for renal tumors are increasing, the conclusions are mixed, especially in terms of postoperative complications, etc, and its safety and efficacy are questionable. We conducted a systematic review and meta-analysis to assess the safety and efficacy of ERAS in the application of partial nephrectomy for renal tumors.

**Methods:**

Pubmed, Embase, Cohrance library, Web of science and Chinese databases (CNKI, VIP, Wangfang and CBM) were systematically searched for all published literature related to the application of enhanced recovery after surgery in partial nephrectomy for renal tumors from the date of establishment to July 15, 2022, and the literature was screened by inclusion/exclusion criteria. The quality of the literature was evaluated for each of the included literature. This Meta-analysis was registered on PROSPERO (CRD42022351038) and data were processed using Review Manager 5.4 and Stata 16.0SE. The results were presented and analyzed by weighted mean difference (WMD), Standard Mean Difference (SMD) and risk ratio (RR) at their 95% confidence interval (CI). Finally, the limitations of this study are analyzed in order to provide a more objective view of the results of this study.

**Results:**

This meta-analysis included 35 literature, including 19 retrospective cohort studies and 16 randomized controlled studies with a total of 3171 patients. The ERAS group was found to exhibit advantages in the following outcome indicators: postoperative hospital stay (WMD=-2.88, 95% CI: -3.71 to -2.05, p<0.001), total hospital stay (WMD=-3.35, 95% CI: -3.73 to -2.97, p<0.001), time to first postoperative bed activity (SMD=-3.80, 95% CI: -4.61 to -2.98, p < 0.001), time to first postoperative anal exhaust (SMD=-1.55, 95% CI: -1.92 to -1.18, p < 0.001), time to first postoperative bowel movement (SMD=-1.52, 95% CI: -2.08 to -0.96, p < 0.001), time to first postoperative food intake (SMD=-3.65, 95% CI: -4.59 to -2.71, p<0.001), time to catheter removal (SMD=-3.69, 95% CI: -4.61 to -2.77, p<0.001), time to drainage tube removal (SMD=-2.77, 95% CI: -3.41 to -2.13, p<0.001), total postoperative complication incidence (RR=0.41, 95% CI: 0.35 to 0.49, p<0.001), postoperative hemorrhage incidence (RR=0.41, 95% CI: 0.26 to 0.66, p<0.001), postoperative urinary leakage incidence (RR=0.27, 95% CI: 0.11 to 0.65, p=0.004), deep vein thrombosis incidence (RR=0.14, 95% CI: 0.06 to 0.36, p<0.001), and hospitalization costs (WMD=-0.82, 95% CI: -1.20 to -0.43, p<0.001).

**Conclusion:**

ERAS is safe and effective in partial nephrectomy of renal tumors. In addition, ERAS can improve the turnover rate of hospital beds, reduce medical costs and improve the utilization rate of medical resources.

**Systematic review registration:**

https://www.crd.york.ac.uk/PROSPERO, identifier CRD42022351038.

## Introduction

Renal tumors are relatively common neoplastic diseases of the urinary system, approximately 85% of which are renal cell carcinomas, and the incidence of the latter is increasing by an average of 0.6% per year (1). It is estimated that 77,410 Chinese will be diagnosed with kidney cancer in China in 2022, and 46,345 of them will die from the disease (2). For renal tumors, the earlier they are detected and treated, the better the prognosis of patients. The traditional treatment for localized renal cell carcinoma is radical nephrectomy, but with the continuous development of medical technology and the improvement of people’s demand for quality of life, the treatment for localized renal cell carcinoma is gradually shifted from radical nephrectomy to partial nephrectomy. Furthermore, the study (3) has shown that compared with radical nephrectomy, partial nephrectomy is more advantageous in terms of postoperative renal function, incidence of chronic kidney disease, tumor recurrence rate, cancer-specific mortality and all-cause mortality, but the complexity of the partial nephrectomy procedure makes it have a higher rate of postoperative complications.

Enhanced recovery after surgery (ERAS) was originally proposed by Danish physicians Wilmore and Kehlet (4) to minimize perioperative physiological dysfunction and surgical stress to promote faster return to normal function, shorter hospital stays and fewer postoperative complications (5). Currently, ERAS has been used in urological procedures such as radical prostatectomy and radical cystectomy, and has shown its advantages (6, 7). Important components of ERAS include detailed preoperative communication, 6-hour preoperative fasting and 2-hour preoperative water fasting, no preoperative enema treatment, preoperative oral carbohydrate, preoperative antibiotic use, intraoperative maintenance of body temperature, intraoperative goal-directed fluid therapy(GDT), reduction of opioid use, early postoperative feeding, early postoperative bed mobility, and early postoperative extubation. One of the more distinctive features of ERAS, GDT, is an optimized intraoperative individualized fluid therapy strategy that uses advanced dynamic detection methods and effective standard treatment procedures to obtain ideal preload and oxygen delivery to improve patient circulation and tissue support, reduce complications, and ultimately achieve a better prognosis. In addition, early bed mobility in ERAS is a way to perform simple activities in bed with the help of a physiotherapist on the day after surgery, and to walk slowly afterwards, which can reduce the complications of prolonged bed rest after surgery and promote rapid postoperative recovery. In recent years, studies on the application of ERAS in partial nephrectomy for renal tumors have been increasing, but the effectiveness of its application in partial nephrectomy for renal tumors is still controversial, especially the findings on postoperative complications are not consistent, so this study was conducted to evaluate the safety and efficacy of ERAS in partial nephrectomy for renal tumors.

## Materials and methods

### Search strategy

The systematic review and meta-analysis strictly followed the preferred reporting items (PRISMA) list for systematic reviews and meta-analyses (8). The systematic evaluation and meta-analysis has been registered on PROSPERO (CRD42022351038).

This search was conducted using subject terms plus free words for all literature related to enhanced recovery after surgery in partial nephrectomy from Pubmed, Embase, Cohrance library, Web of science, CNKI, VIP, Wangfang, and CBM from the date of library construction to July 15, 2022. There are no publication language and time restrictions in the search process. In addition, references to the literature were retroactively included to supplement access to relevant literature. Search terms include: “enhanced recovery after surgery”, “fast track surgery”, “ERAS”, “FTS”, “renal Cell Carcinomas”, “Kidney Neoplasms”, “partial nephrectomy”, “nephron sparing surgery”, etc. Take Pubmed search strategy as an example, see **Table 1**.

**Table 1:** Search strategy of PubMed.

Number Search Strategy
#1 (enhanced recovery after surgery[MeSH]) OR (Enhanced Postsurgical Recovery[Title/Abstract]) OR (Postsurgical Recoveries, Enhanced[Title/Abstract]) OR (Postsurgical Recovery, Enhanced[Title/Abstract]) OR (Recovery, Enhanced Postsurgical[Title/Abstract]) OR (ERAS[Title/Abstract]) OR (fast track surgery[Title/Abstract]) OR (fast track[Title/Abstract]) OR (FTS[Title/Abstract]) OR (enhanced recovery[Title/Abstract]) OR (enhanced recovery program[Title/Abstract]) OR (early recovery after surgery[Title/Abstract]) OR (accelerated recovery from surgery[Title/Abstract]) OR (fast rehabilitation[Title/Abstract]) OR (accelerated rehabilitation[Title/Abstract]) OR (multimodal perioperative care[Title/Abstract])
#2 (Carcinomas, Renal Cell[Mesh])OR(Renal Cell Carcinomas[Title/Abstract])OR(Nephroid Carcinoma[Title/Abstract])OR(Carcinoma, Nephroid[Title/Abstract])OR(Nephroid Carcinomas[Title/Abstract])OR(Adenocarcinoma Of Kidney[Title/Abstract])OR(Adenocarcinoma Of Kidneys[Title/Abstract])OR(Kidney, Adenocarcinoma Of[Title/Abstract])OR(Renal Cell Carcinoma[Title/Abstract])OR(Renal Cell Cancer[Title/Abstract])OR(Cancer, Renal Cell[Title/Abstract])OR(Renal Cell Cancers[Title/Abstract])OR(Adenocarcinoma, Renal[Title/Abstract])OR(Renal Adenocarcinoma[Title/Abstract])OR(Renal Adenocarcinomas[Title/Abstract])OR(Renal Carcinoma[Title/Abstract])OR(Carcinoma, Renal[Title/Abstract])OR(Renal Carcinomas[Title/Abstract])OR(Adenocarcinoma, Renal Cell[Title/Abstract])OR(Adenocarcinomas, Renal Cell[Title/Abstract])OR(Renal Cell Adenocarcinoma[Title/Abstract])OR(Renal Cell Adenocarcinomas[Title/Abstract])OR(Chromophobe Renal Cell Carcinoma[Title/Abstract])OR(Sarcomatoid Renal Cell Carcinoma[Title/Abstract])OR(Papillary Renal Cell Carcinoma[Title/Abstract])OR(Renal Cell Carcinoma, Papillary[Title/Abstract])OR(Chromophil Renal Cell Carcinoma[Title/Abstract])OR(Clear Cell Renal Cell Carcinoma[Title/Abstract])OR(Grawitz Tumor[Title/Abstract])OR(Tumor, Grawitz[Title/Abstract])OR(Clear Cell Renal Carcinoma[Title/Abstract])OR(Carcinoma, Hypernephroid[Title/Abstract])OR(Hypernephroid Carcinoma[Title/Abstract])OR(Hypernephroid Carcinomas[Title/Abstract])OR(Hypernephroma[Title/Abstract])OR(Hypernephromas[Title/Abstract])OR(Collecting Duct Carcinoma (Kidney) [Title/Abstract])OR(Carcinoma, Collecting Duct (Kidney) [Title/Abstract])OR(Carcinomas, Collecting Duct (Kidney) [Title/Abstract])OR(Collecting Duct Carcinomas (Kidney) [Title/Abstract])OR(Collecting Duct Carcinoma of the Kidney[Title/Abstract])OR(Renal Collecting Duct Carcinoma[Title/Abstract])OR(Collecting Duct Carcinoma[Title/Abstract])OR(Carcinoma, Collecting Duct[Title/Abstract])OR(Carcinomas, Collecting Duct[Title/Abstract])OR(Collecting Duct Carcinomas[Title/Abstract]) OR (Kidney Neoplasms[Mesh]) OR (Kidney Neoplasm[Title/Abstract])OR (Neoplasm, Kidney[Title/Abstract])OR (Renal Neoplasms[Title/Abstract])OR (Neoplasm, Renal[Title/Abstract])OR (Neoplasms, Renal[Title/Abstract])OR (Renal Neoplasm[Title/Abstract])OR (Neoplasms, Kidney[Title/Abstract])OR (Cancer of Kidney[Title/Abstract])OR (Kidney Cancers[Title/Abstract])OR (Renal Cancer[Title/Abstract])OR (Cancer, Renal[Title/Abstract])OR (Cancers, Renal[Title/Abstract])OR (Renal Cancers[Title/Abstract])OR (Cancer of the Kidney[Title/Abstract])OR (Kidney Cancer[Title/Abstract])OR (Cancer, Kidney[Title/Abstract])OR (Cancers, Kidney[Title/Abstract])
#3 (partial nephrectomy[Title/Abstract])OR (renal tumor resection[Title/Abstract])OR (nephron sparing surgery[Title/Abstract]) OR (partial kidney resection[Title/Abstract])OR (nephrectomy, partial[Title/Abstract])
#4 #2 OR #3
#5 #1 AND #4

### Inclusion and exclusion criteria

Inclusion criteria: (1) Patients with renal tumors who underwent partial nephrectomy; (2) A comparative study between ERAS and conventional nursing; (3) At least one primary or secondary outcome must be included: length of postoperative hospital stay(from the end of surgery to the time of discharge), total hospital stay(time from admission to discharge), time to first postoperative bed activity, time to first postoperative anal exhaust, time to first postoperative bowel movement, time to first postoperative food intake, time to removal of catheter, time to removal of drainage tube, incidence of total postoperative complications, and hospitalization cost.

Exclusion criteria: (1) Literature with completely duplicated or mostly duplicated data sources; (2) Literature that is not available in full text; (3) Literature that does not identify the type of surgery or non-partial nephrectomy; (4) Literature with ERAS of less than 2 factors; (5) Types of articles such as overviews, case reports, reviews, etc; (6) NOS < 6.

### Literature screening and data extraction

The literature searched from each database was imported into EndNote X7 and screened independently by two authors (WWJ and LTY) according to the inclusion exclusion criteria, with the assistance of a third author (MXQ) to judge if disagreement arose. For literature screening, duplicates were first screened out and excluded, then apparently irrelevant studies were excluded by reading the titles and abstracts of the literature, and finally the full text was read to determine the final literature to be included in this study. The content to be extracted included: first author, year of publication, study type, country, age, gender, sample size, composition of ERAS, surgical modality (robot-assisted laparoscopic surgery, laparoscopic surgery, or open surgery), surgical access (transabdominal or extra-abdominal), and outcome indicators (primary outcome: postoperative length of stay and total length of stay; secondary outcomes: postoperative time to first bed activity, postoperative time to first anal exhaust, postoperative time to first bowel movement, postoperative time to first food intake, catheter removal time, drainage tube removal time, total postoperative complication rate, and hospitalization cost).

### Literature quality assessment

Two authors jointly conducted quality assessment of randomized controlled studies (RCTs) according to the Cochrane Risk of Bias tool (9) and case-control studies or cohort studies according to the Newcastle-Ottawa (NOS) scale (10). The Cochrane Risk of Bias Tool assessment focused on whether the random allocation method was correct, whether the allocation scheme was well concealed, whether blinding was implemented, whether the data results were complete, whether there was selective reporting, and whether there were other biases in the study. The NOS scale was evaluated for appropriateness of cohort selection (0-4 points), comparability between groups (0-2 points), and method of determining outcomes of interest (0-3 points), for a total of 9 points.

### Statistical analysis

This Meta-analysis was performed using Review Manager 5.4 and Stata 16.0SE. For dichotomous variables, the relative risk ratio (RR) is used as an effect indicator; for continuous variables, the weighted mean difference (WMD) or standardized mean difference (SMD) is used as an effect indicator, and estimates and 95% confidence intervals (95% CI) are provided. The chi-square test and I^2^ test were used to assess heterogeneity between studies. If there was no statistical heterogeneity between studies (p≥0.10 and I²≤50%), the fixed-effects model was used; conversely, the random-effects model was used. Subgroup analysis was used to analyze the source of heterogeneity. Sensitivity analysis was used to test the stability of the meta-analysis results. The presence of publication bias was assessed using funnel plots and Egger’s test.

## Results

### Literature search results and characteristics of eligible studies

**Figure 1:**
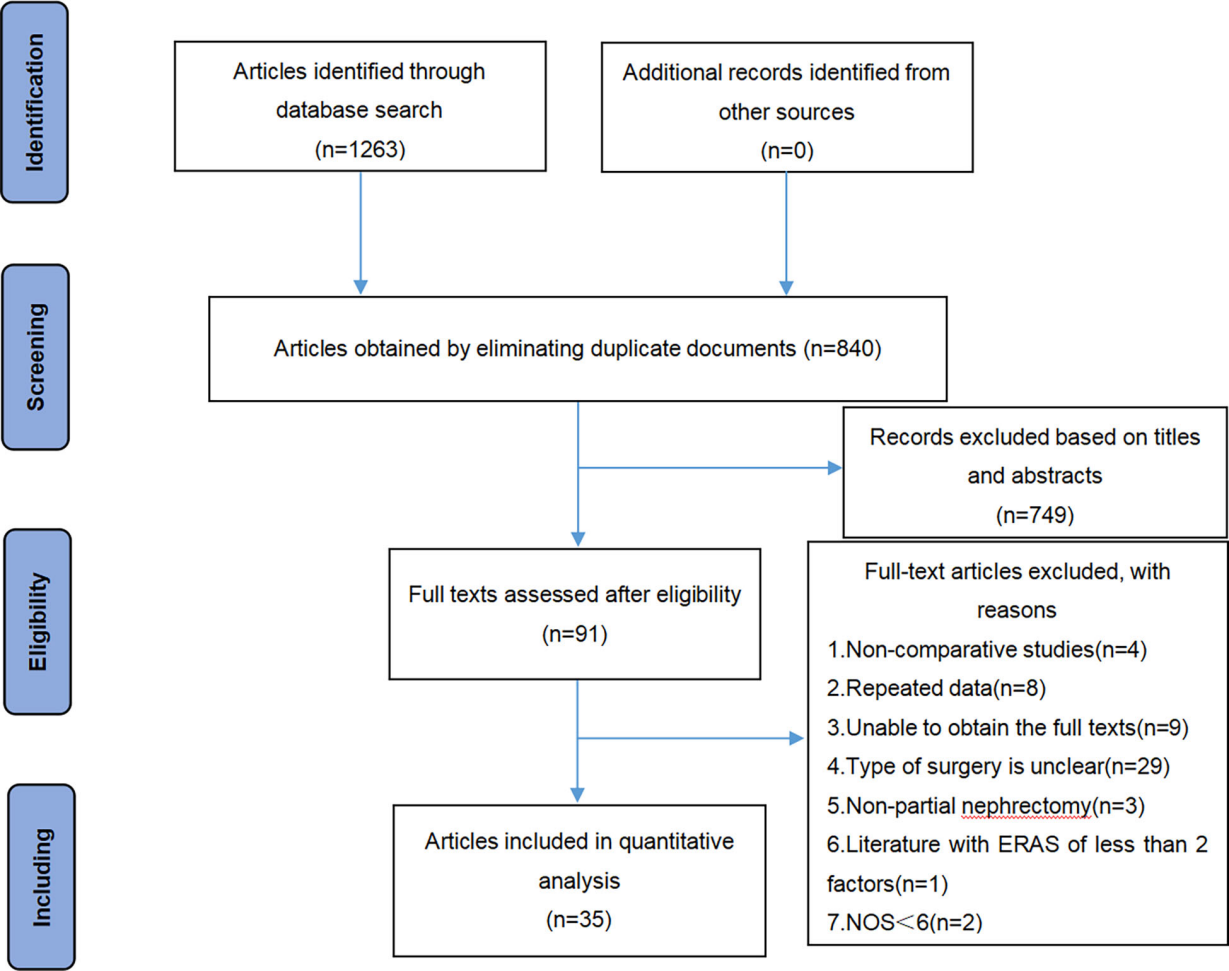
Flow chart of PRISMA for literature screening.

A total of 1263 associated articles were searched, and 35 articles were finally obtained through screening (11–45), of which 19 were retrospective cohort studies and 16 were RCTs. A total of 3171 patients were involved in the 35 literature, with 1632 patients in the ERAS group and 1539 patients in the usual care group. The literature screening process is shown in **Figure 1**. The basic characteristics of the included literature are shown in **Table 2**. The ERAS elements included in the studies are shown in **Table 3**.

**Table 2:** Basic characteristics of the included literature.

Study	Year	Country	Study design	Age(years)		Gender(Male/Female)		Sample size		Surgery method	Surgery Approach	outcomes
				ERAS	RC	ERAS	RC	ERAS	RC			
Zhao (11)	2019	China	RCS	51.38 ± 4.62	50.71 ± 4.72	45/29	46/28	74	74	NA	NA	A C D E F I
Gong (12)	2019	China	RCT	55.05 ± 12.55	52.59 ± 10.49	25/17	22/19	42	41	RAPN	NA	A D E F G J
Chen (13)	2019	China	RCT	63.2 ± 3.7	63.4 ± 3.8	30/13	29/14	43	43	LPN	NA	A C D
Zhou (14)	2021	China	RCS	49.52 ± 4.53	47.57 ± 4.48	14/11	13/12	25	25	NA	NA	A D E G H I
Wu (15)	2019	China	RCT	44.67 ± 2.13	42.19 ± 2.01	26/31	25/32	57	57	RAPN	RS	A I J
Huang (16)	2017	China	RCS	52.4 ± 6.2	53.6 ± 6.1	13/11	12/9	24	21	RAPN	NA	A G H I
Guo (17)	2020	China	RCS	53 ± 13.88	53 ± 13.90	28/22	27/24	50	51	LPN	RS	A B C D G H J
Shi (18)	2021	China	RCS	53.55 ± 12.44	55.92 ± 13.15	35/20	31/19	55	50	LPN	RS/AC	A C D F G H I
Liu (19)	2017	China	RCS	55.5 ± 9.1	53.5 ± 11.4	19/16	19/13	35	32	LPN	NA	A C D J
Zhao (20)	2017	China	RCS	56.3 ± 4.2	55.8 ± 3.6	10/17	15/13	27	28	LPN	RS	A C D F G H I J
Wang (21)	2019	China	RCS	45.1 ± 9.7	43.2 ± 10.0	15/12	17/12	29	27	LPN	NA	A C D I J
Zhong (22)	2020	China	RCS	60.2 ± 10.8	49.5 ± 11.5	22/8	24/6	30	30	RAPN	NA	A D I
Wang (23)	2017	China	RCS	46.5 ± 23.5	47.4 ± 23.4	18/12	17/13	30	30	LPN	RS	A C D F G H J
Zeng (24)	2017	China	RCT	51.3 ± 3.8	52.1 ± 4.3	17/12	14/14	29	28	LPN	NA	A C D I J
Gong (25)	2018	China	RCS	NA	NA	NA	NA	28	27	LPN	RS	A D F G H J
Tu (26)	2019	China	RCT	50.24 ± 9.02	46.75 ± 8.64	31/29	38/22	60	60	LPN	NA	A C D F I
Xue (27)	2015	China	RCS	NA	NA	NA	NA	35	35	LPN	RS	A C D F G H I
Cheng (28)	2012	China	RCS	21~65	27~75	25/10	23/12	35	35	LPN	NA	B D E I
Song (29)	2012	China	RCT	50 ± 0.7	48.9 ± 0.5	28/17	27/17	45	44	LPN	NA	A G I
Yao (30)	2018	China	RCT	53.42 ± 13.24	52.37 ± 12.48	36/21	34/23	57	57	LPN	RS	A C D F H I
Hu (31)	2018	China	RCS	47.7 ± 6.6	48.9 ± 7.3	23/27	28/22	50	50	RAPN	RS	A C E F H I J
Liao (32)	2018	China	RCT	51.23 ± 3.68	52.13 ± 3.52	23/16	25/14	39	39	OPN	NA	A C D F G H I J
Yang (33)	2021	China	RCT	52.98 ± 13.552	50.00 ± 12.406	24/16	19/21	40	40	LPN	NA	A F I
Ruan (34)	2022	China	RCS	53.7 ± 12.8	51.7 ± 14.7	40/18	41/12	58	53	LPN	NA	B C E F G H I
Gu (35)	2020	China	RCT	49.04 ± 5.38	48.35 ± 5.74	21/13	18/16	34	34	RAPN	NA	A C D G H I
Huang (36)	2020	China	RCT	54.6 ± 8.6	54.8 ± 7.2	13/12	14/11	25	25	LPN	RS	A C D F G I
He (37)	2017	China	RCT	58.24 ± 12.26	58.43 ± 11.82	20/31	23/28	51	51	LPN	NA	I
Liu (38)	2019	China	RCT	55.70 ± 9.32	54.95 ± 10.08	14/16	15/15	30	30	LPN	NA	A C D F G
Kang (39)	2019	China	RCT	53.2	49.6	22/18	24/16	40	40	LPN	NA	A C D F G H I
Lin (40)	2018	China	RCS	56.5 ± 5.2	55.8 ± 5.6	41/28	54/24	69	78	LPN	RS	A C D F G H I J
Miao (41)	2020	China	RCT	53.41 ± 14.25	55.63 ± 13.79	81/29	74/29	110	103	LPN	NA	A B D E G H I J
Inès Dominique (42)	2021	France	RCS	58.2 ± 1.28	60.2 ± 1.85	77/35	38/12	112	50	RAPN	NA	I
Xue (43)	2022	China	RCT	53.70 ± 10.69	55.97 ± 12.39	21/10	20/9	31	29	LPN	NA	A C D F J
Bilal Chughtai (44)	2008	United States	RCS	39~73	32~74	22/11	18/7	33	25	OPN	NA	I
Nosov, A. K (45)	2019	Russia	RCS	56.93 ± 12.75	56.65 ± 10.38	NA	NA	100	97	OPN	RS	I

RCS, Retrospective cohort study; RCT, Randomized controlled trials; ERAS, enhanced recovery after surgery; RC, Routine Care; RAPN, Robot-assisted partial nephrectomy; LPN, Laparoscopic partial nephrectomy; OPN, Open partial nephrectomy; RS, Retroperitoneal space; NA, Not available; A, Post-operative hospitalization time; B, Total length of stay; C, First time out of bed after surgery; D, Time of first postoperative anal exhaust; E, Time of first bowel movement after surgery; F, Time of first postoperative food intake; G, Drainage tube removal time;H, Removal time of catheter; I, Total postoperative complications; J, Hospitalization costs.

**Table 3:** ERAS elements included in the studies.

Study	Year	A	B	C	D	E	F	G	H	I	J	K	L	
Zhao (11)	2019	√		√		√	√		√	√	√	√		8
Gong (12)	2019	√	√	√	√		√	√	√	√	√	√		10
Chen (13)	2019	√								√				2
Zhou (14)	2021	√	√	√	√		√	√	√	√	√	√	√	11
Wu (15)	2019	√	√		√				√	√	√			6
Huang (16)	2017	√			√					√	√	√	√	6
Guo (17)	2020	√	√	√	√		√	√	√	√	√	√	√	11
Shi (18)	2021	√	√	√	√		√	√	√	√	√	√	√	11
Liu (19)	2017	√	√	√	√		√	√	√	√	√		√	10
Zhao (20)	2017	√	√	√	√		√	√		√	√	√	√	10
Wang (21)	2019		√	√	√				√	√	√	√		7
Zhong (22)	2020	√							√	√	√			4
Wang (23)	2017	√	√	√	√		√		√	√	√	√	√	10
Zeng (24)	2017	√	√	√	√		√		√	√	√	√	√	10
Gong (25)	2018	√	√	√	√		√	√	√	√	√	√	√	11
Tu (26)	2019	√							√	√	√			4
Xue (27)	2015	√	√	√	√		√	√	√	√	√	√	√	11
Cheng (28)	2012	√	√	√	√		√	√	√	√	√		√	10
Song (29)	2012	√			√		√	√		√	√	√	√	8
Yao (30)	2018	√	√	√		√	√				√			6
Hu (31)	2018	√	√	√	√		√			√	√		√	8
Liao (32)	2018	√	√	√	√		√		√	√	√	√	√	10
Yang (33)	2021	√	√	√	√	√	√	√	√	√	√	√	√	12
Ruan (34)	2022	√		√	√		√	√	√	√	√	√	√	10
Gu (35)	2020	√			√				√	√	√	√	√	7
Huang (36)	2020	√	√	√	√		√			√	√	√	√	9
He (37)	2017	√	√	√	√	√	√	√	√	√	√	√	√	12
Liu (38)	2019	√								√	√	√		4
Kang (39)	2019	√	√	√	√	√	√		√	√	√	√	√	11
Lin (40)	2018	√	√	√	√		√	√		√	√	√	√	10
Miao (41)	2020	√	√	√	√		√	√	√	√	√	√	√	11
Inès Dominique (42)	2021	√	√		√				√	√	√	√		7
Xue (43)	2022	√	√	√	√			√	√	√	√	√	√	12
Bilal Chughtai (44)	2008	√				√			√	√	√	√	√	7
Nosov, A. K (45)	2019	√	√			√			√					3

A, Pre-admission detailed education, consultation and communication; B, 6h preoperative fasting, 2h preoperative water fasting; C, No enema treatment required; D, Pre-operative oral carbohydrate; E, Preoperative antibiotic use; F, Intraoperative insulation; G, Intraoperative goal-directed fluid therapy; H, Use of non-opioid pain relievers; I, Early postoperative feeding; J, Early postoperative bed mobility; K, Early postoperative removal of drainage tubes; L, Early postoperative removal of catheter; √, The paper has this content.

### Literature quality assessment results

**Figure 2:**
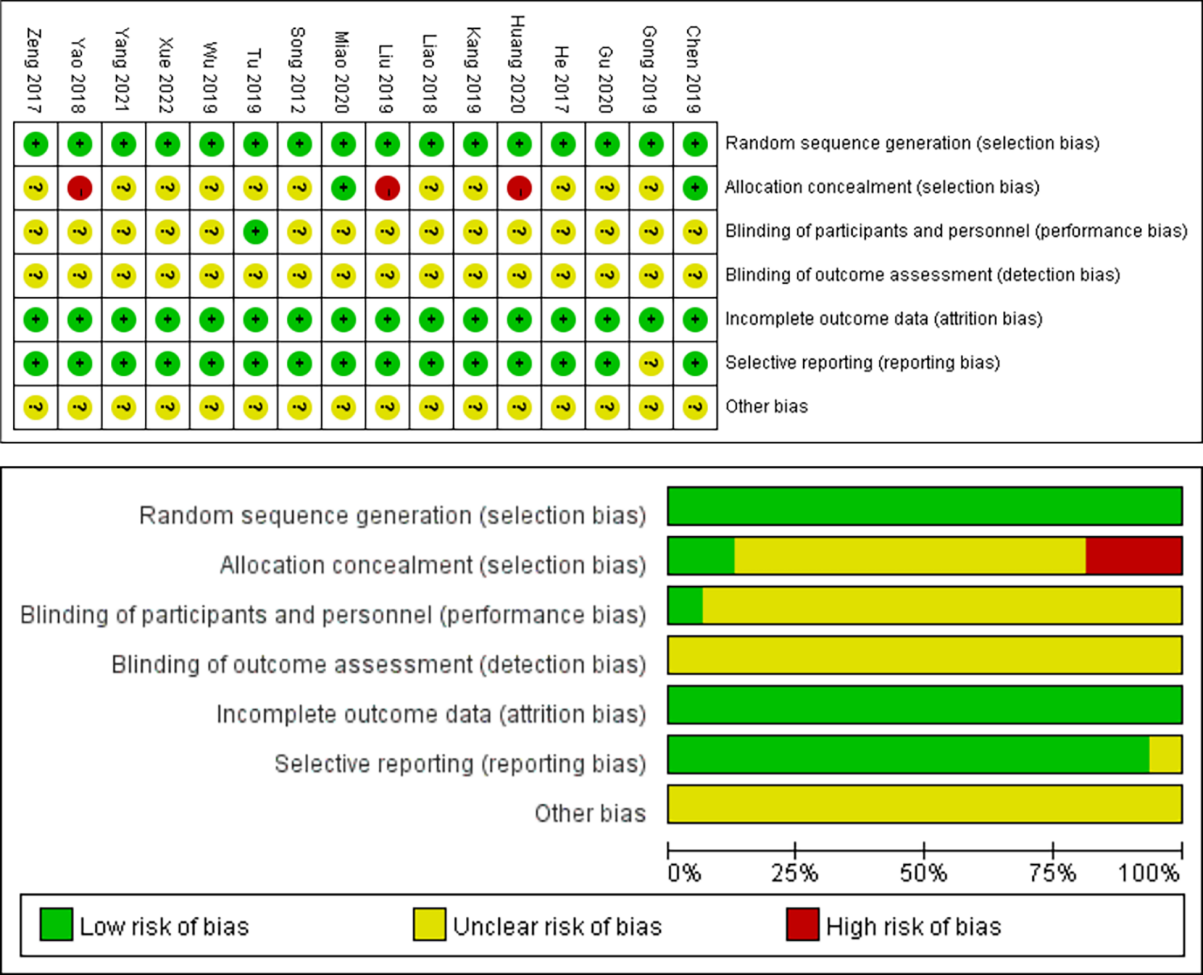
Quality assessment of RCT.

The quality of the included retrospective cohort studies was assessed using the NOS scale, which showed six with a score of 6, six with a score of 7, one with a score of 8, and six with a score of 9. The detailed scoring results are shown in **Table 4**. Quality assessment of randomized controlled studies using the Cochrane Risk of Bias tool identified three different studies (30, 36, 38) that were at high risk of selection bias. See **Figure 2**.

**Table 4:** Quality assessment of retrospective cohort studies.

Study	Year	A	B	C	D	E	F	G	H	I
Zhao (11)	2019	1	1	1	1	1	1	0	0	6
Zhou (14)	2021	1	1	1	1	1	1	0	0	6
Huang (16)	2017	1	1	1	1	1	1	0	0	6
Guo (17)	2020	1	1	1	1	2	1	0	1	8
Shi (18)	2021	1	1	1	1	2	1	1	1	9
Liu (19)	2017	1	1	1	1	1	1	0	0	6
Zhao (20)	2017	1	1	1	1	2	1	1	1	9
Wang (21)	2019	1	1	1	1	2	1	0	0	7
Zhong (22)	2020	1	1	1	1	1	1	0	0	6
Wang (23)	2017	1	1	1	1	2	1	0	0	7
Gong (25)	2018	1	1	1	1	1	1	0	0	6
Xue (27)	2015	1	1	1	1	2	1	0	0	7
Cheng (28)	2012	1	1	1	1	2	1	0	0	7
Hu (31)	2018	1	1	1	1	2	1	0	0	7
Ruan (34)	2022	1	1	1	1	2	1	1	1	9
Lin (40)	2018	1	1	1	1	2	1	1	1	9
Inès Dominique (42)	2021	1	1	1	1	2	1	1	1	9
Bilal Chughtai (44)	2008	1	1	1	1	2	1	1	1	9
Nosov, A. K (45)	2019	1	1	1	1	2	1	0	0	7

A, Exposure queue representation; B, Selection of non-exposed queues; C, Determination of exposure; D, No subject had an outcome event prior to study entry; E, Comparability of exposed and non-exposed queues; F, Ending measurement method; G, Adequacy of follow-up visits; H, Is the follow-up visit complete; I, NOS scores.

## Meta-analysis results

### Primary outcomes

#### Postoperative hospitalization time

**Figure 3:**
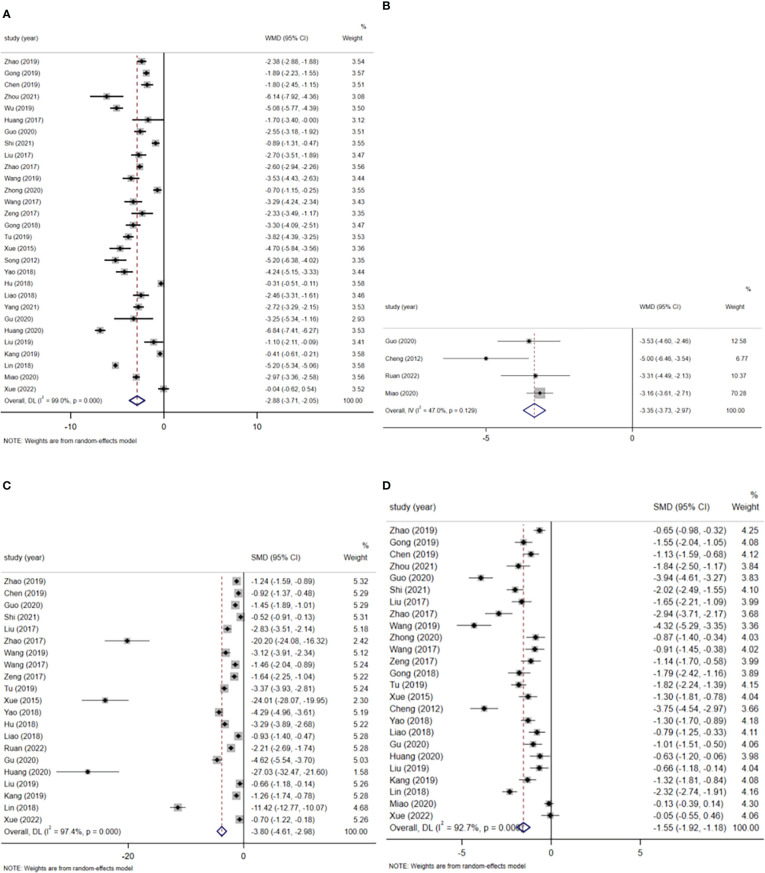
**(A)** Postoperative hospital stay. **(B)** Total hospital time. **(C)** First time out of bed after surgery. **(D)** Time of first postoperative anal exhaust.

A total of 29 studies reported on postoperative length of stay, involving a total of 2471 patients (1243 patients in the ERAS group and 1228 patients in the routine care group). A random-effects model summary showed that the postoperative hospital stay was significantly shorter by 2.88 days in the ERAS group compared with the routine care group (WMD=-2.88, 95% CI: -3.71 to -2.05, p<0.001, I^2 ^= 99.0%) (**Figure 3A**). Subgroup analysis by surgical approach showed that the postoperative hospital stay was reduced by 2.09 days (WMD=-2.09, 95% CI: -3.37 to -0.80, p<0.001, I^2 ^= 97.7%) in the subgroup of robot-assisted laparoscopic surgery (**Figure 1** in **Supplementary Data Sheet 1**); the postoperative hospital stay was reduced by 3.00 days in the subgroup of laparoscopic surgery (WMD=-3.00, 95% CI: -4.03 to -1.98, p < 0.001, I^2^ = 99.0%) (**Figure 1** in **Supplementary Data Sheet 1**).

#### Total hospitalization time

Four studies reported total length of stay, involving a total of 495 patients (253 patients in the ERAS group and 242 patients in the routine care group). A fixed-effects model summary showed that the total length of stay was 3.35 days shorter in the ERAS group compared with the routine care group (WMD=-3.35, 95% CI: -3.73 to -2.97, p<0.001, I^2 ^= 47.0%) (**Figure 3B**).

### Secondary outcomes

#### First time out of bed after surgery

A total of 21 studies reported time to first postoperative bed activity. A total of 1794 patients were involved, including 900 patients in the ERAS group. A random-effects model summary showed that the ERAS group significantly moved patients out of bed earlier after surgery compared to the routine care group (SMD=-3.80, 95% CI: -4.61 to -2.98, p<0.001, I^2 ^= 97.4%) (**Figure 3C**). Subgroup analysis by surgical approach showed that ERAS significantly earlier postoperative bed mobility in both robot-assisted laparoscopic surgery and laparoscopic surgery (robot-assisted laparoscopic surgery: SMD=-3.91, 95% CI: -5.21 to -2.61, p<0.001, I^2 ^= 82.2%; laparoscopic surgery: SMD=-4.33, 95% CI. -5.33 to -3.33, p<0.001, I^2 ^= 97.7%) (**Figure 2** in **Supplementary Data Sheet 1**).

#### Time of first postoperative anal exhaust

A total of 25 studies reported the time of first postoperative anal exhaust and included a total of 2114 patients (1062 patients in the ERAS group and 1052 patients in the usual care group). The Meta-analysis showed that the ERAS group had an earlier time to first postoperative anal exhaust compared to the usual care group (SMD=-1.55, 95% CI: -1.92 to -1.18, p<0.001, I^2 ^= 92.7%) (**Figure 3D**). Subgroup analysis by surgical approach showed that ERAS resulted in earlier postoperative anal exhaust in patients who underwent robotic-assisted laparoscopic surgery and laparoscopic surgery (robotic-assisted laparoscopic surgery: SMD=-1.15, 95% CI: -1.56 to -0.74, p<0.001, I^2 ^= 48.7%; laparoscopic surgery: SMD=-1.70, 95% CI: - 2.18 to -1.23, p<0.001, I^2 ^= 94.1%) (**Figure 3** in **Supplementary Data Sheet 1**).

#### Time of first bowel movement after surgery

**Figure 4:**
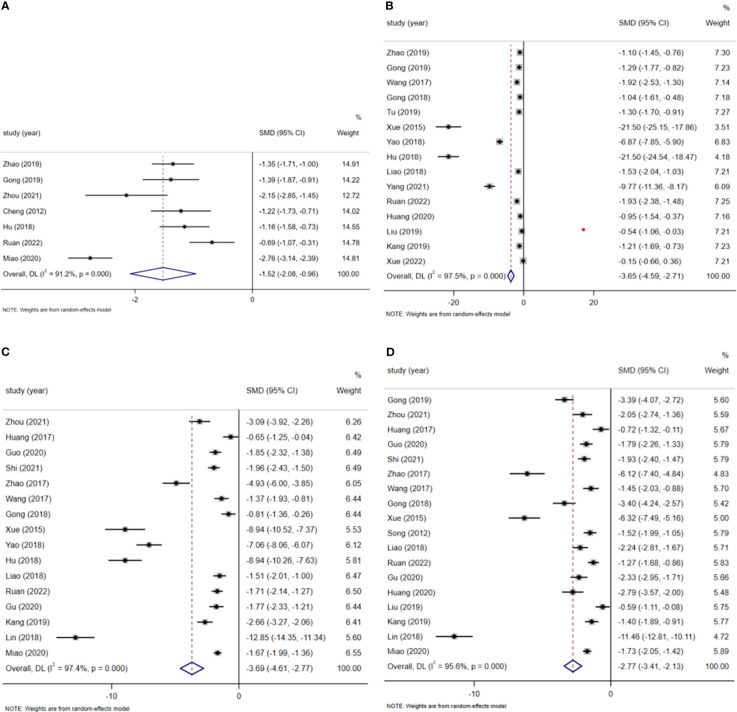
**(A)** Time of first bowel movement after surgery. **(B)** Time of first postoperative food intake. **(C)** Removal time of catheter. **(D)** Removal time of drainage tube.

A total of 7 studies reported the time to first postoperative bowel movement. A total of 775 patients were included, including 394 patients in the ERAS group and 381 patients in the usual care group. A random-effects model summary showed that ERAS significantly earlier the time to first postoperative bowel movement in patients compared to routine care (SMD=-1.52, 95% CI: -2.08 to -0.96, p<0.001, I^2 ^= 91.2%) (**Figure 4A**). Subgroup analysis by surgical approach showed that ERAS was associated with earlier postoperative defecation in both robot-assisted laparoscopic surgery and laparoscopic surgery (robot-assisted laparoscopic surgery: SMD=-1.26, 95% CI: -1.58 to -0.94, p<0.001, I^2 ^= 0.0%; laparoscopic surgery: SMD=-1.56, 95% CI: - 2.89 to -0.23, p=0.021, I^2 ^= 96.7%) (**Figure 4** in **Supplementary Data Sheet 1**).

#### Time of first postoperative food intake

A total of 15 studies reported the time to first postoperative food intake, involving a total of 1269 patients (639 patients in the ERAS group and 630 patients in the routine care group). A random-effects model summary showed patients in the ERAS group had their first postoperative meal earlier compared to the usual care group (SMD=-3.65, 95% CI: -4.59 to -2.71, p<0.001, I^2 ^= 97.5%) (**Figure 4B**). Subgroup analysis by surgical approach showed no significant difference in time to first postoperative food intake with ERAS in robotic-assisted laparoscopic surgery (SMD=-11.34, 95% CI: -31.15 to 8.47, p=0.262, I^2 ^= 99.4%), but limited studies were included and the results need to be treated with caution; ERAS in laparoscopic surgery was able to advance patients’ postoperative time to first meal (SMD=-3.37, 95% CI: -4.50 to -2.24, p<0.001, I^2 ^= 97.4%) (**Figure 5** in **Supplementary Data Sheet 1**).

**Figure 5:**
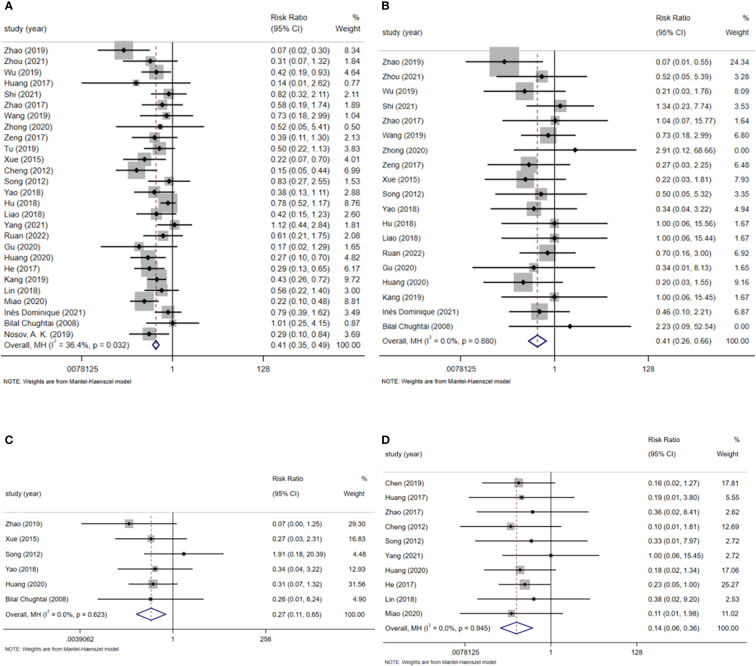
**(A)** Postoperative complications. **(B)** Postoperative bleeding. **(C)** Postoperative urine leakage. **(D)** Deep vein thrombosis.

#### Removal time of catheter

A total of 16 studies reported validated data on catheter removal time, containing 1453 patients (731 patients in the ERAS group and 722 patients in the routine care group). The meta-analysis showed that the ERAS group had a shorter catheter removal time compared to the usual care group (SMD=-3.69, 95% CI: -4.61 to -2.77, p<0.001, I^2 ^= 97.4%) (**Figure 4C**). Subgroup analysis by surgical approach showed ERAS reduced catheter removal time in both robot-assisted laparoscopic and laparoscopic procedures (robot-assisted laparoscopic surgery: SMD=-3.72, 95% CI: -7.10 to -0.33, p=0.031, I^2 ^= 98.4%; laparoscopic surgery: SMD=-3.98, 95% CI: - 5.12 to -2.84, p<0.001, I^2 ^= 97.7%) (**Figure 6** in **Supplementary Data Sheet 1**).

**Figure 6:**
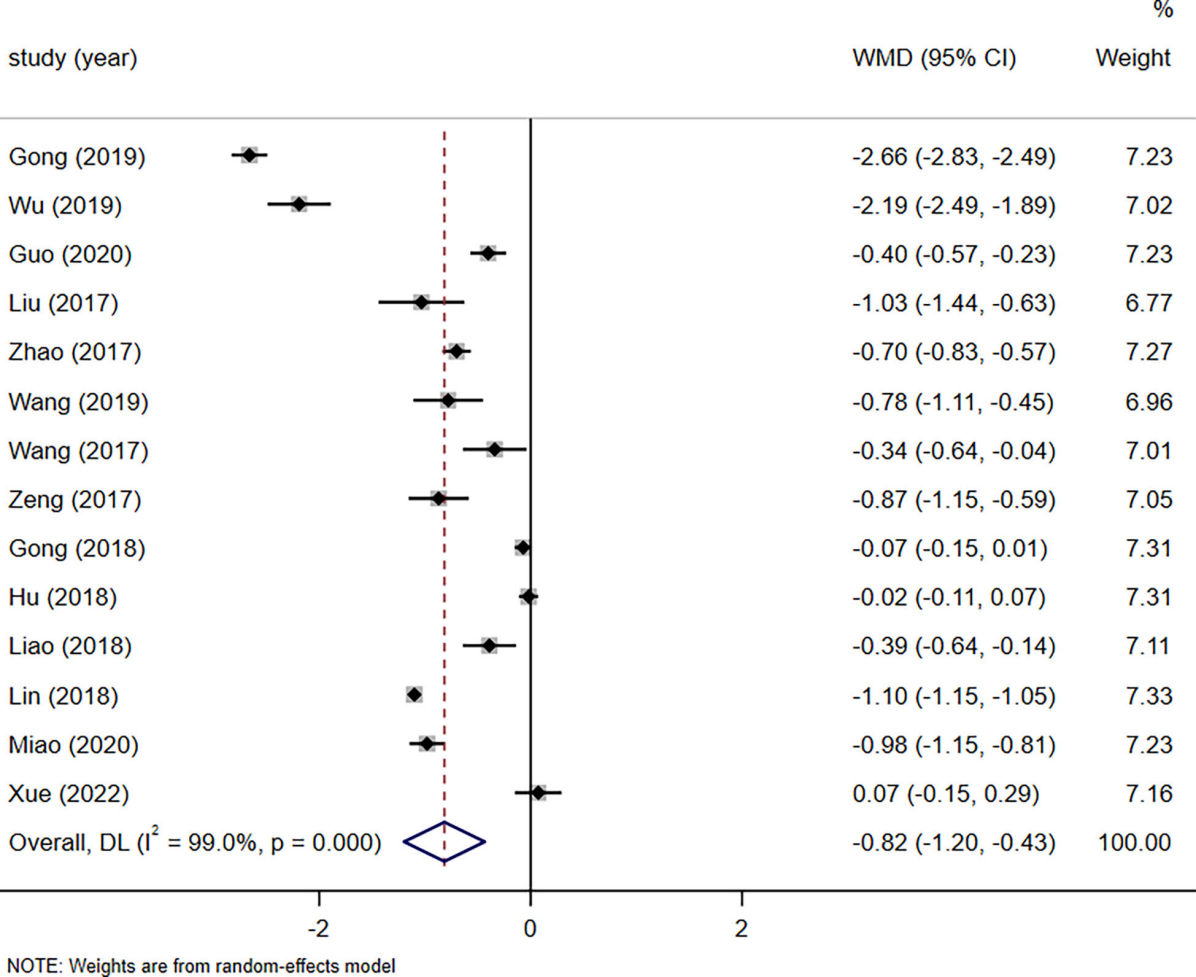
Impact of ERAS on patient hospitalization costs.

#### Removal time of drainage tube

A total of 18 studies containing valid data on drain removal time involved a total of 1521 patients (766 patients in the ERAS group and 755 patients in the routine care group). In a random effects model pooled analysis, patients in the ERAS group had a shorter drain removal time compared to those in the usual care group (SMD=-2.77, 95% CI: -3.41 to -2.13, p<0.001, I^2 ^= 95.6%) (**Figure 4D**). In a subgroup analysis by surgical method, ERAS was able to reduce the removal time of drains in patients both in robot-assisted laparoscopic surgery and in laparoscopic surgery (robot-assisted laparoscopic surgery: SMD=-2.14, 95% CI: -3.66 to -0.62, p=0.006, I^2 ^= 94.2%; laparoscopic surgery: SMD=-3.05, 95% CI: -3.88 to -2.22, p<0.001, I^2 ^= 96.6%) (**Figure 7** in **Supplementary Data Sheet 1**).

### Total postoperative complications

A total of 27 studies reported total postoperative complications, mainly including fever, nausea and vomiting, abdominal distention, postoperative hemorrhage, postoperative urinary leakage, and deep vein thrombosis, involving a total of 2599 patients, (1343 patients in the ERAS group and 1256 patients in the usual care group). In a fixed-effects model pooled analysis, ERAS reduced the rate of total postoperative complications after partial nephrectomy compared to usual care (RR=0.41, 95% CI: 0.35-0.49, p<0.001, I^2 ^= 36.4%) (**Figure 5A**). In a subgroup analysis by surgical approach, ERAS was demonstrated to reduce total postoperative complication rates in both robot-assisted laparoscopic surgery, laparoscopic surgery and open surgery (robot-assisted laparoscopic surgery: RR=0.61, 95% CI: 0.45-0.84, p=0.002, I^2 ^= 6.0%; laparoscopic surgery: RR=0.40, 95% CI: 0.32~ 0.49, p<0.001, I^2 ^= 20.8%; open surgery: RR=0.42, 95% CI: 0.22-0.82, p=0.010, I^2 ^= 0.0%) (**Figure 8** in **Supplementary Data Sheet 1**).

### Postoperative hemorrhage

A total of 20 studies reported postoperative hemorrhage involving a total of 1670 patients (878 patients in the ERAS group and 792 patients in the routine care group) and a total of 87 patients (26 patients in the ERAS group and 61 patients in the routine care group) with postoperative hemorrhage. A fixed-effects model summary showed ERAS was more able to reduce the incidence of postoperative hemorrhage in patients compared to usual care (RR=0.41, 95% CI: 0.26 to 0.66, p<0.001, I^2 ^= 0.0%) (**Figure 5B**).

### Postoperative urine leakage

A total of 6 studies reported postoperative urinary leakage involving 529 patients (269 patients in the ERAS group and 260 patients in the routine care group), of which a total of 30 patients had postoperative leakage (6 patients in the ERAS group and 24 patients in the routine care group). In a fixed-effects model pooled analysis, ERAS reduced the incidence of postoperative urinary leakage in patients compared to routine care (RR=0.27, 95% CI: 0.11 to 0.65, p=0.004, I^2 ^= 0.0%) (**Figure 5C**).

### Deep vein thrombosis

A total of 11 studies reported the incidence of deep vein thrombosis in a total of 993 patients (498 patients in the ERAS group and 495 patients in the routine care group), of which 44 patients developed deep limb venous thrombosis (5 patients in the ERAS group and 39 patients in the routine care group). A fixed-effects model summary showed ERAS was more able to reduce the formation of DVT in patients compared to usual care (RR=0.14, 95% CI: 0.06-0.36, p<0.001, I^2 ^= 0.0%) (**Figure 5D**).

### Hospitalization costs

A total of 14 studies reported patient hospital costs for a total of 1247 patients (626 patients in the ERAS group and 621 patients in the routine care group). A random-effects model pooled analysis showed that ERAS reduced patients’ hospital costs compared to usual care (WMD=-0.82, 95% CI: -1.20 to -0.43, p<0.001, I^2 ^= 99.0%) (**Figure 6**). A subgroup analysis by surgical approach found that ERAS reduced patient hospitalization costs in both robot-assisted laparoscopic and laparoscopic procedures (robot-assisted laparoscopic surgery: WMD=-2.44, 95% CI: -2.90 to -1.98, p=0.006, I^2 ^= 86.2%; laparoscopic surgery: WMD=-0.56, 95% CI: - 0.90 to -0.22, p=0.001, I^2 ^= 98.6%) (**Figure 9** in **Supplementary Data Sheet 1**).

### Sensitivity analysis

Sensitivity analysis was performed on the outcome indicators of postoperative hospital stay, total hospital stay, first time out of bed after surgery, time of first postoperative anal exhaust, time of first bowel movement after surgery, time of first postoperative food intake, removal time of catheter, removal time of drainage tube, incidence of total postoperative complications, incidence of postoperative hemorrhage, incidence of postoperative urine leakage, incidence of deep vein thrombosis, and hospitalization costs by excluding one study at a time and observing the combined effect size. The results of the sensitivity analysis showed that the pooled results of these outcome indicators were stable. See **Supplementary Data Sheet 2**.

### Publication bias

Publication bias for each outcome indicator was assessed using funnel plots with Egger’s test. The funnel diagram of each outcome indicator is shown in **Supplementary Data Sheet 3**. Egger’s test showed significant publication bias for first time out of bed after surgery (P<0.001), time of first postoperative anal exhaust (P<0.001), time of first postoperative food intake (P<0.001), removal time of catheter (P<0.001), and removal time of drainage tube (P<0.001), and significant publication bias for postoperative hospital stay (P=0.881), total hospital time (P=0.233), time to first postoperative bowel movement (P=0.907), total postoperative complication rate (P=0.049), postoperative hemorrhage rate (P=0.554), postoperative urine leakage rate (P=0.853), deep vein thrombosis rate (P=0.614) and hospitalization cost (P=0.940) were not publication biased.

## Discussion

ERAS is a new model of care based on evidence-based medicine, consisting of surgery, nursing, anesthesia, rehabilitation, nutrition, and other disciplines, which aims to reduce the length of hospital stay and postoperative complications by reducing the stress response caused by surgical trauma, thereby facilitating rapid patient recovery (46). The effectiveness of ERAS was initially demonstrated in colorectal surgery and then widely used in urology for radical cystectomy and radical prostatectomy with favorable results. Although ERAS has been well studied in partial nephrectomy for renal tumors, the conclusions are mixed, especially in terms of postoperative complications and other aspects, and its safety and efficacy are debatable. This study was the first to compare the recent efficacy of ERAS and routine care application in the perioperative period of partial nephrectomy for renal tumors by Meta-analysis method to confirm that ERAS application is safe and effective in partial nephrectomy for renal tumors.

Postoperative length of stay and total length of stay are important indicators to assess the effectiveness of ERAS. The results of this study showed that both the subgroup analysis and the pooled analysis of the robot-assisted laparoscopic surgery group and the laparoscopic surgery group showed that the postoperative hospital stay was significantly shorter in the ERAS group than in the usual care group (WMD=-2.88, 95% CI: -3.71 to -2.05, p<0.001), which is consistent with the study of Domenic Di Rollo et al (47). Similarly, ERAS reduced the total length of stay (WMD=-3.35, 95% CI: -3.73 to -2.97, p<0.001), but given the limited number of included studies, no subgroup analysis was performed. The reduction of postoperative hospital stay may be closely related to preoperative oral carbohydrates, preoperative prehabilitation, effective postoperative analgesia, early feeding, and early bed mobility treatment. The absence of preoperative enema treatment reduces intestinal flora and water and electrolyte disturbances, which in turn accelerates the recovery of intestinal function (48). With regard to postoperative feeding time in the robot-assisted laparoscopic subgroup, however, there was no statistical difference between the ERAS group and the usual care group (P=0.262). Nevertheless, considering the small number of included studies and the fact that ERAS was able to shorten the feeding time after robotic-assisted partial nephrectomy in all included studies (12, 31), it cannot be completely stated that ERAS cannot shorten the feeding time after robotic-assisted partial nephrectomy, and further clinical validation is required for follow-up. This study showed that ERAS can shorten the removal time of catheter and drainage tube (P < 0.001), which can indirectly reduce the occurrence of catheter-related urinary tract infections (49, 50) and also facilitate early patient mobility. However, there was publication bias in the results of catheter and drainage tube removal times, which was considered to be caused by the small sample size of some included literature and unreported outcome indicators (14, 16, 20, 25, 36), so further validation in future clinical studies with large samples should be performed.

The incidence of total postoperative complications is an important indicator to assess the safety of ERAS. The main postoperative complications of partial nephrectomy include fever, nausea and vomiting, abdominal distention, postoperative hemorrhage, postoperative urinary leakage, and deep vein thrombosis, among other complications. The results of this study showed that the implementation of ERAS in either the robotic-assisted laparoscopic group or the laparoscopic group did not increase the postoperative complication rate, but rather led to a decrease (RR=0.41, 95% CI: 0.35 to 0.49, p<0.001). This may be related to ERAS elements such as oral intake of liquid carbohydrates and goal-directed fluid management 2 hours prior to surgery. Perioperative application of goal-directed fluid management significantly reduces postoperative complications, shortens the length of hospital stay, and accelerates patient recovery (51, 52). Hemorrhage and urinary leakage are the two main procedure-related renal complications of partial nephrectomy (53), and studies have shown that ERAS reduces the incidence of both complications, which is associated with detailed preoperative counseling and preoperative oral carbohydrates (54). Deep vein thrombosis is a serious life-threatening complication, and with its development associated with tumors, stress reactions due to surgical trauma, prolonged postoperative bed rest, and other risk factors. In the case of ERAS, it was able to reduce the stress response caused by the procedure and enable patients to get out of bed earlier, which in turn reduced the formation of deep vein thrombosis. In terms of medical costs of partial nephrectomy, both in China and in other countries they mainly include: medical care, nursing care, medication, blood, laboratory, radiology, inspection, pathology, housing, disposable material, anesthesia and operating room use. Compared to other countries, medical care and nursing care costs are lower in China, while disposable material and medication costs are higher, probably because disposable supplies and medications are mainly imported into China. A study from China showed that disposable consumables and drugs accounted for the largest share of hospitalization costs (55). ERAS can further reduce the use of drugs and disposable supplies by facilitating rapid postoperative recovery and reducing surgical complications, thereby reducing the cost of hospitalization for patients. This study likewise showed that ERAS did not increase patients’ hospital costs, but instead reduced them by 0.82 million RMB (WMD = -0.82, 95% CI: -1.20 to -0.43, p < 0.001), which, in addition to the previously stated reasons, was closely associated with a shorter hospital stay (56). Therefore, ERAS is very helpful for patients with renal tumors requiring partial nephrectomy worldwide, especially those coming from low- and middle-income countries.

The strengths of this study are the search of eight major databases to ensure that no relevant articles were missed, the inclusion of a relatively large number of randomized controlled trials and a large sample size, and the fact that the pooled results were shown to be stable after sensitivity analysis, which provides reliable evidence for the results of this study. Nevertheless, there are some limitations in this study. In the first place, most of the included studies were from China, and the findings could not be well generalized to other regions, probably because the implementation of care for partial nephrectomy in China was performed in the hospital, whereas in other regions, care continued in the community with early discharge (57). Therefore, more high-quality randomized controlled studies are still needed worldwide. As the second, because the outcome indicators such as time to first postoperative bed activity, time to first postoperative anal exhaust, time to first postoperative bowel movement, time to first postoperative meal, time to drainage tube removal, and time to catheter removal in the included literature were not in consistent units, the standardized mean difference (SMD) was used as the effect indicator, making the data results only provide qualitative clinical significance. The third one is that the heterogeneity of the outcome indicators, except for total postoperative complications, postoperative hemorrhage, postoperative leakage, and deep vein thrombosis, was too high and remained high after subgroup analysis, which was considered to be caused by various factors such as study type, surgical approach, and surgical access. Finally, publication bias in time to first postoperative bed activity, time to first postoperative anal exhaust, time to first postoperative feeding, time to catheter removal, and time to drainage tube removal may be due to a combination of small sample sizes in some studies, unavailability of data from some studies, failure of some studies to report the above outcome indicators, duplication of publication in national and international journals by some authors, and the fact that negative results are not easily published.

## Conclusion

ERAS is safe and effective in partial nephrectomy of renal tumors. In addition, ERAS can improve the turnover rate of hospital beds, reduce medical costs and improve the utilization rate of medical resources.

## Data availability statement

The original contributions presented in the study are included in the article/**Supplementary Material**. Further inquiries can be directed to the corresponding author.

## Author contributions

WW, LT, and MX contributed to conception and design of the study. ZD organized the database. WW, LT, and MX performed the statistical analysis. WW wrote the first draft of the manuscript. ZC, WC, DZ, JT, and ZF wrote sections of the manuscript. All authors contributed to manuscript revision, read, and approved the submitted version.
