# Supplementary material for: Application of enhanced recovery after surgery in partial nephrectomy for renal tumors: A systematic review and meta-analysis
Source: Front Oncol. 2023 Feb 9;13:1049294. doi: 10.3389/fonc.2023.1049294 (PMC9947501; doi:10.3389/fonc.2023.1049294)
Supplement: Supplementary file 1 [file DataSheet_1.docx]

**Subgroup analysis**


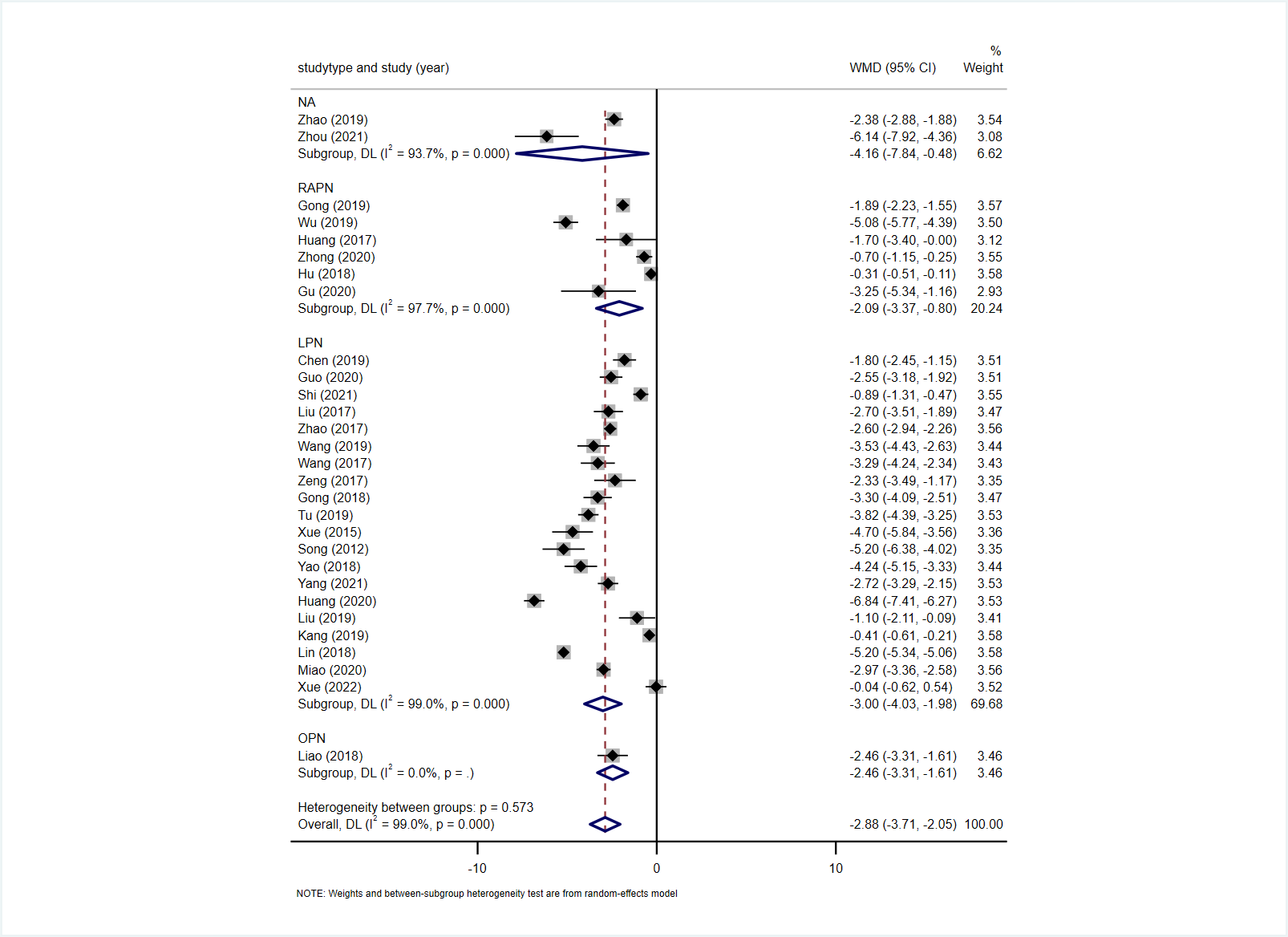


**Figure 1. Postoperative hospital stay.**


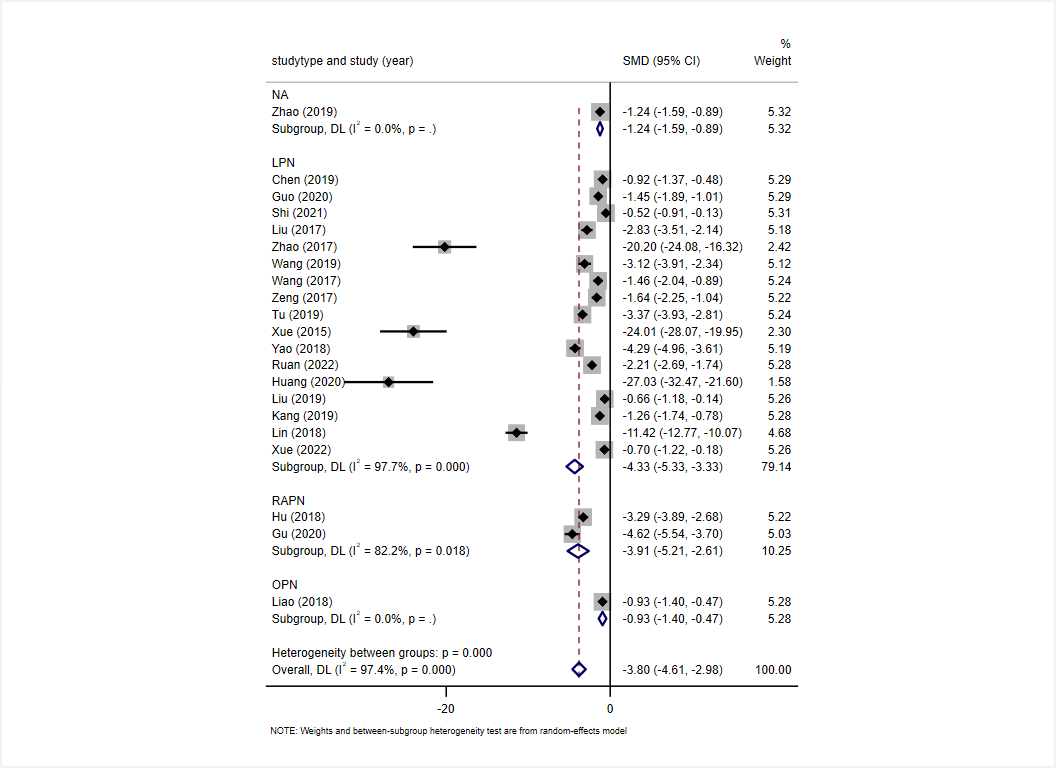


**Figure 2. First time out of bed after surgery.**


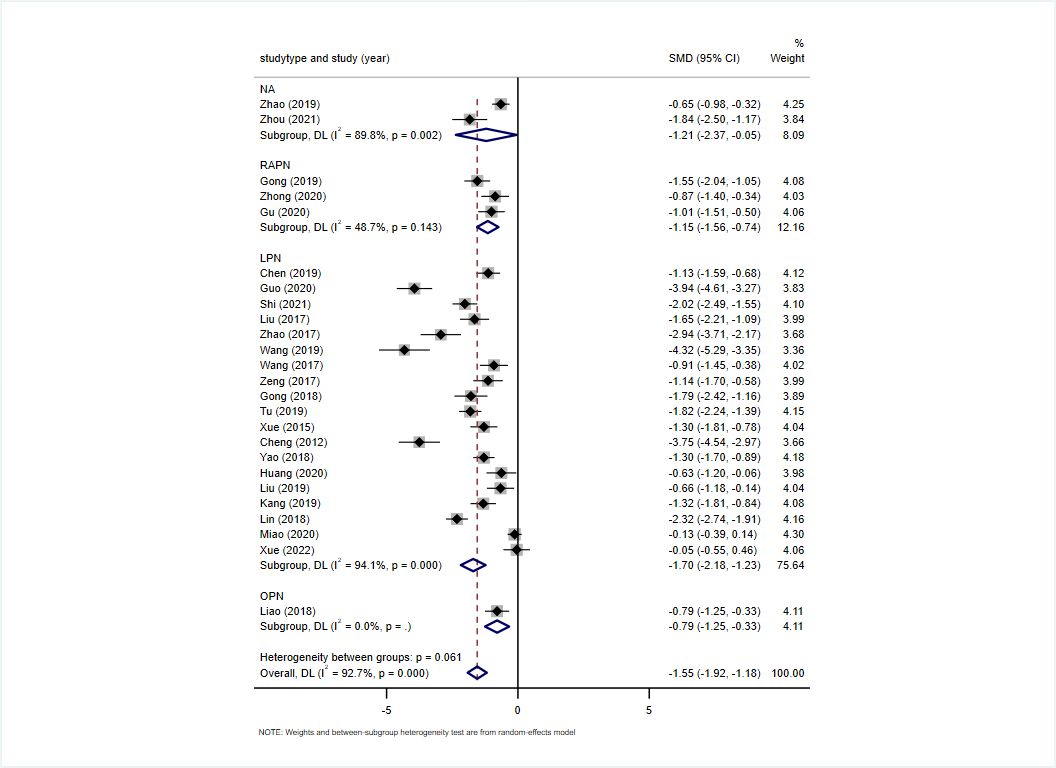


**Figure 3. Time of first postoperative anal exhaust.**


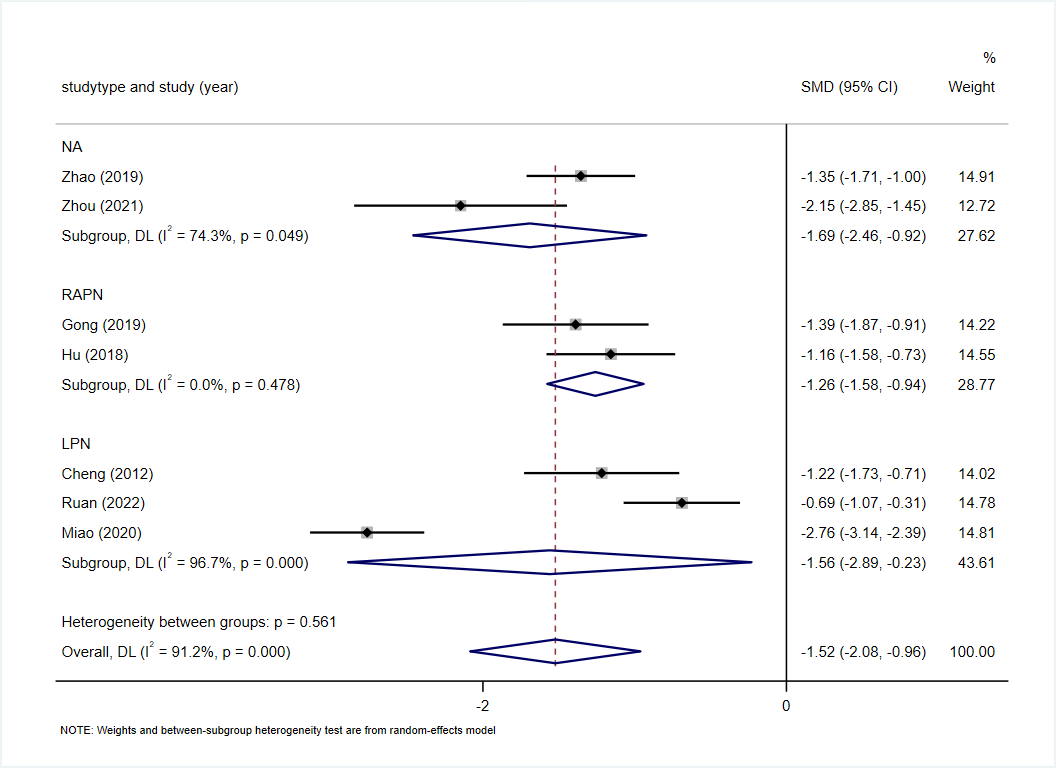


**Figure 4. Time of first bowel movement after surgery.**


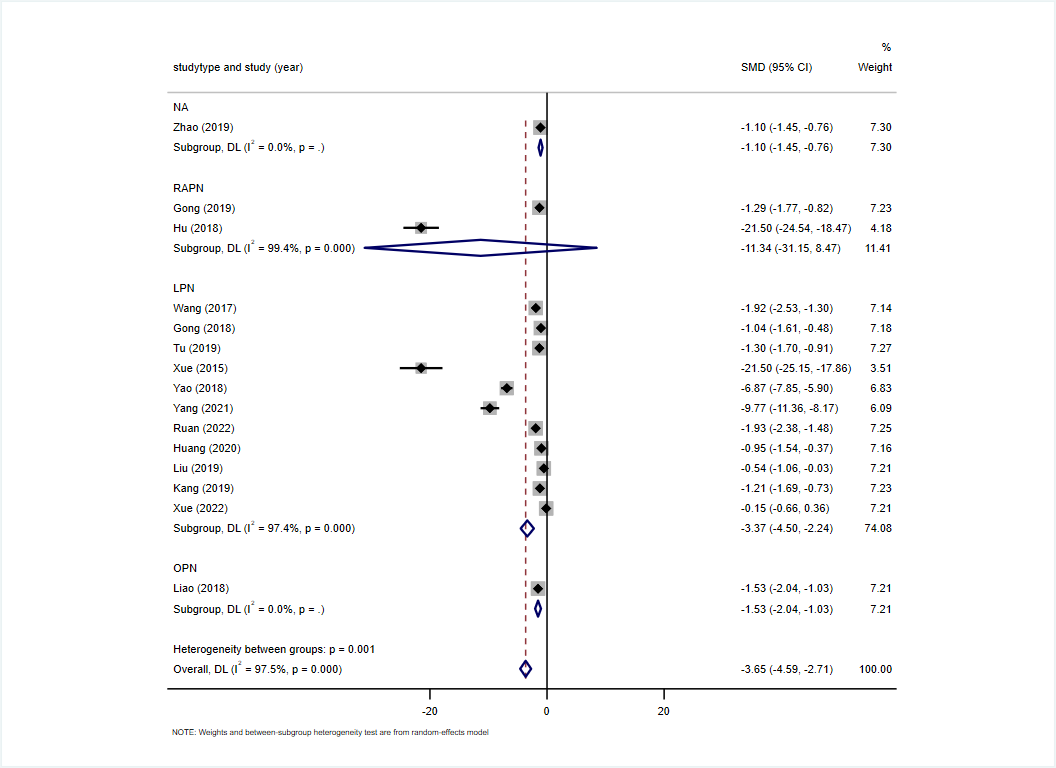


**Figure 5. Time of first postoperative food intake.**


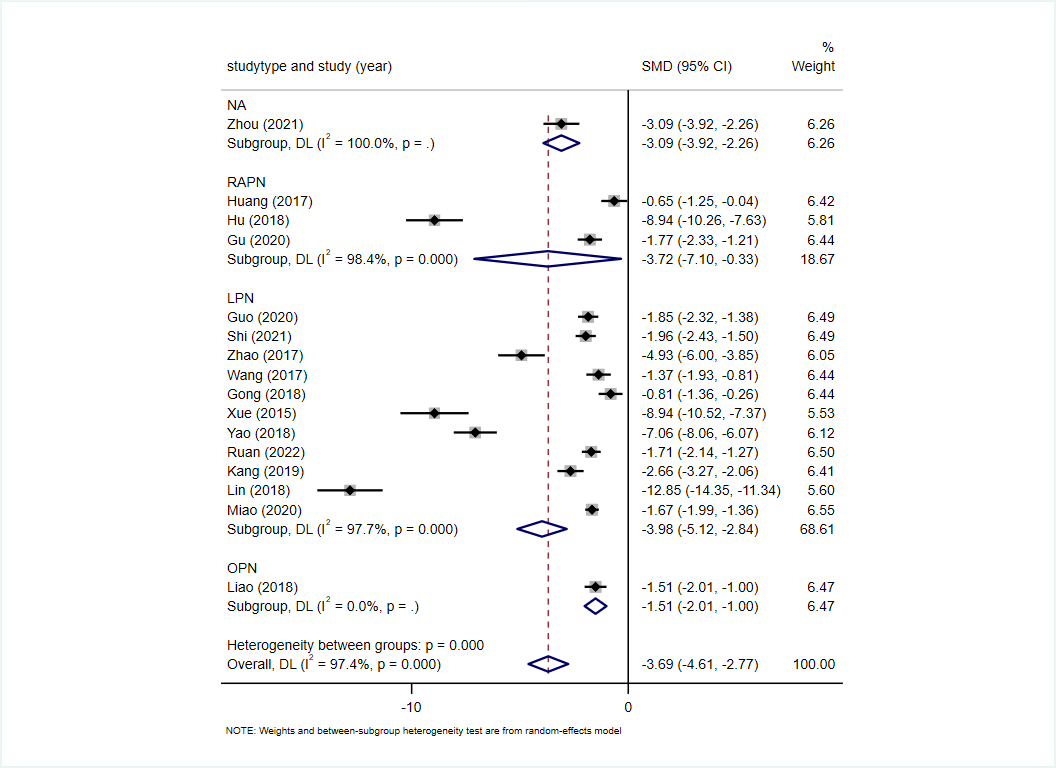


**Figure 6. Removal time of catheter.**


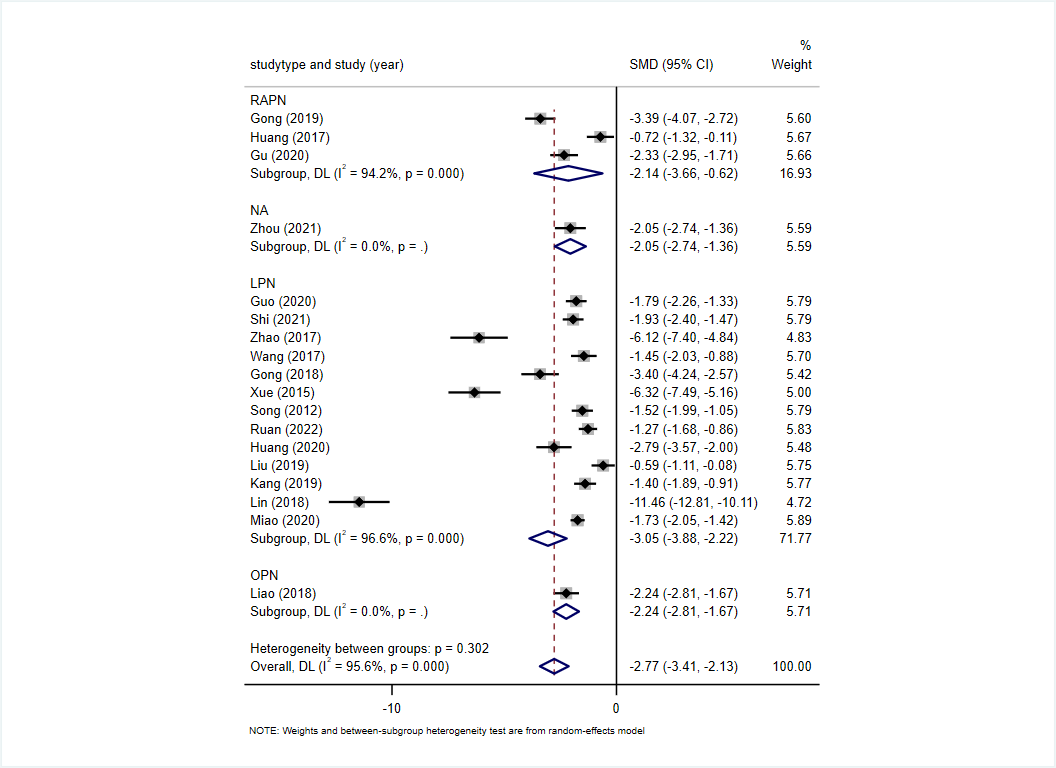


**Figure 7. Removal time of drainage tube.**


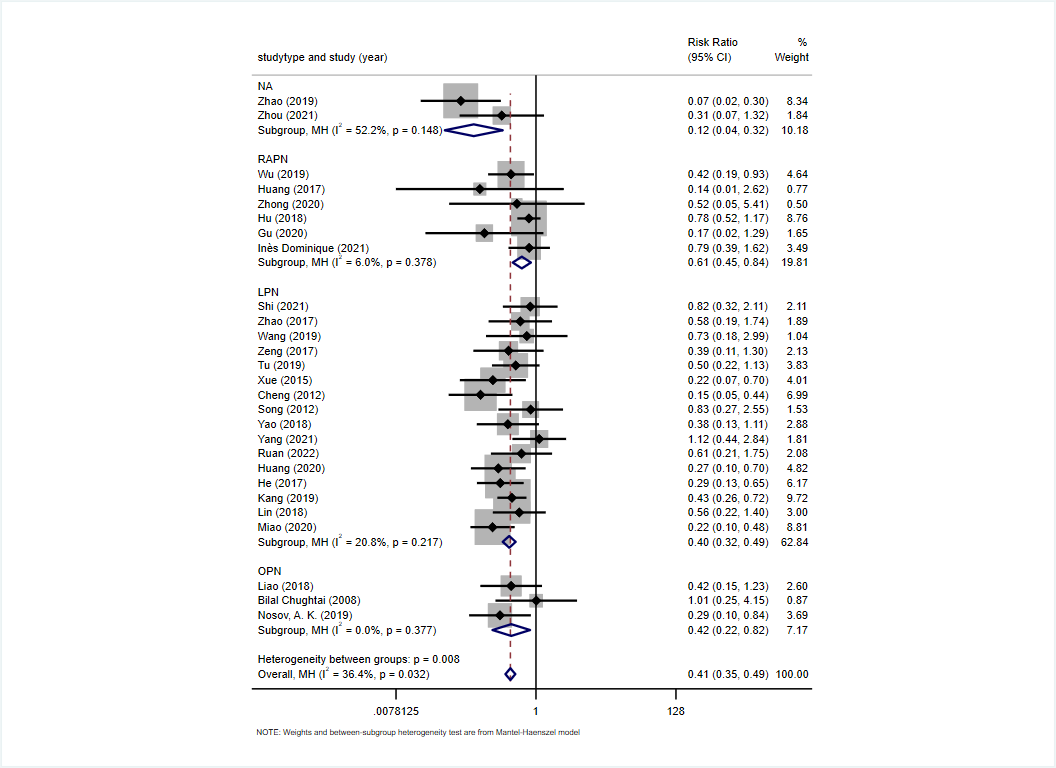


**Figure 8. Total postoperative complications.**


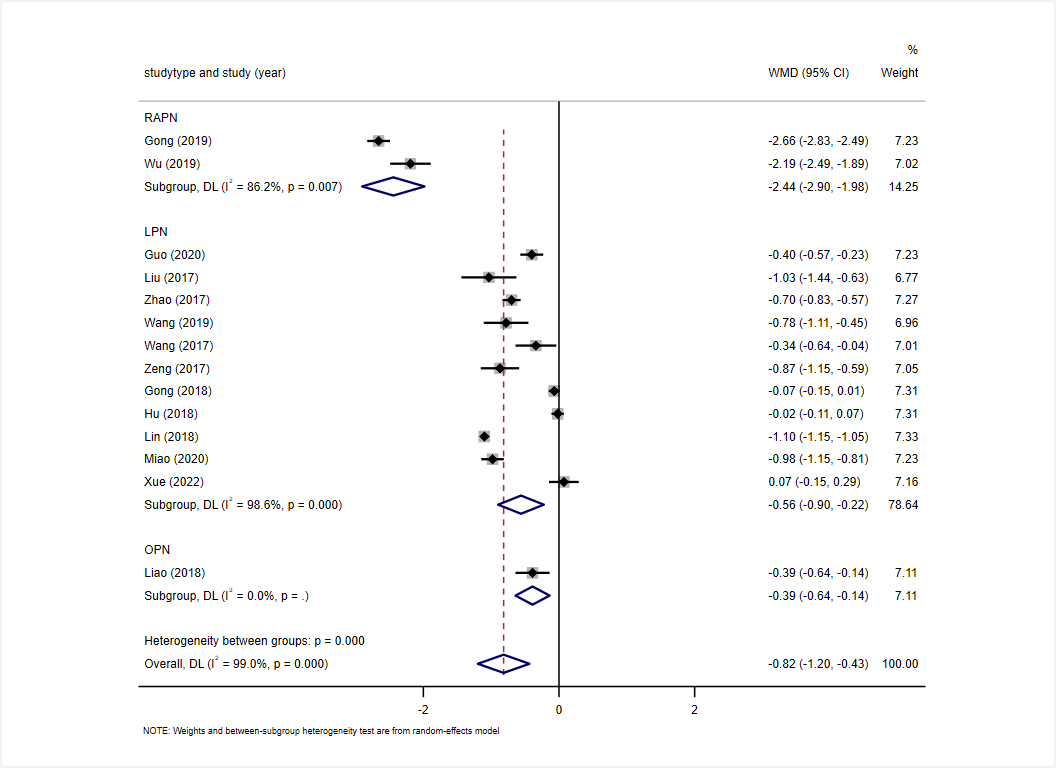


**Figure 9. Hospitalization costs.**
