# Supplementary material for: Application of enhanced recovery after surgery in partial nephrectomy for renal tumors: A systematic review and meta-analysis
Source: Front Oncol. 2023 Feb 9;13:1049294. doi: 10.3389/fonc.2023.1049294 (PMC9947501; doi:10.3389/fonc.2023.1049294)
Supplement: Supplementary file 3 [file DataSheet_3.docx]

**Funnel plots of publication bias**


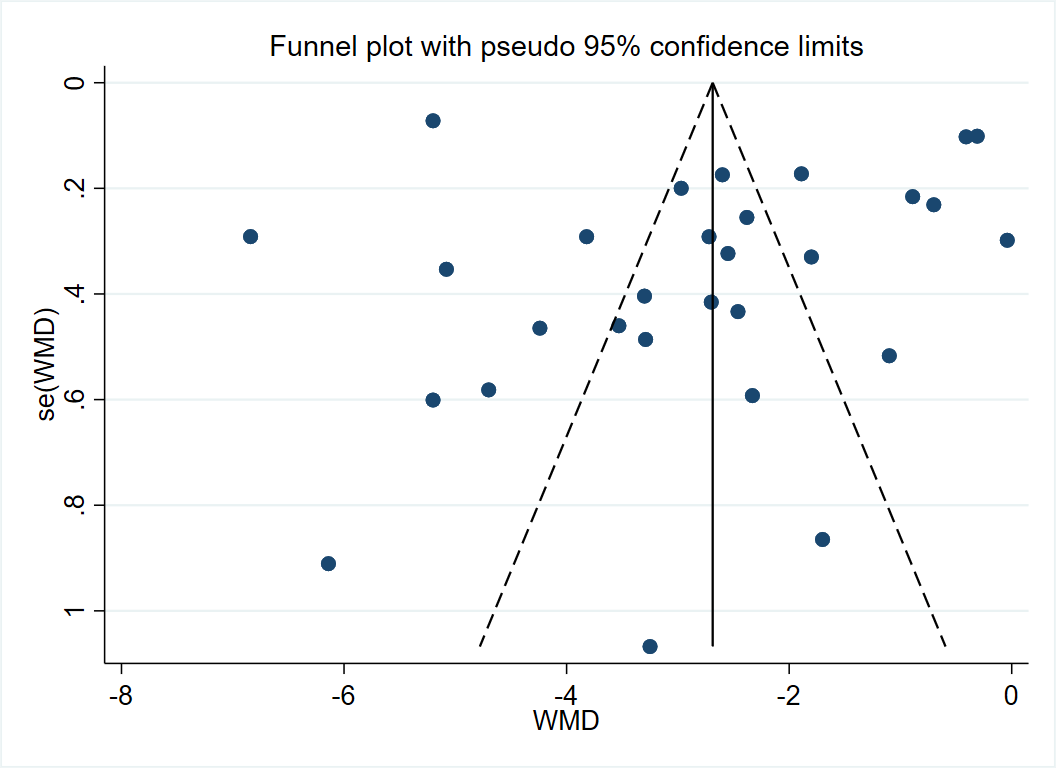


**Figure 1 Postoperative hospital stay**


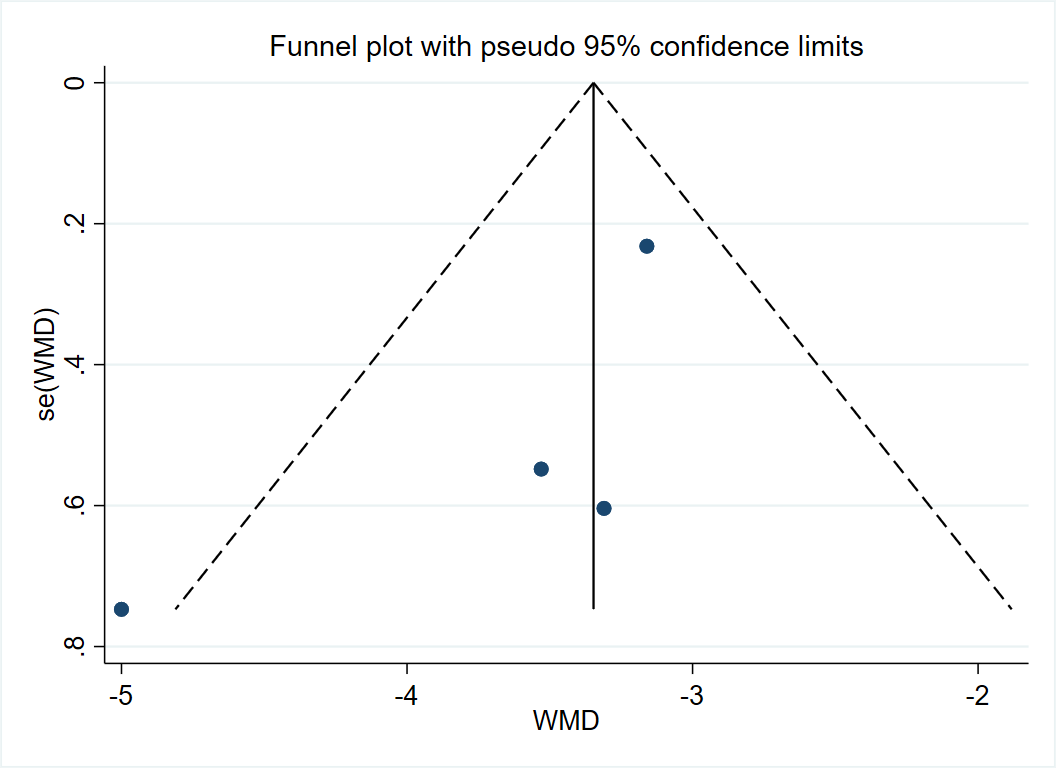


**Figure 2 Total hospital time**


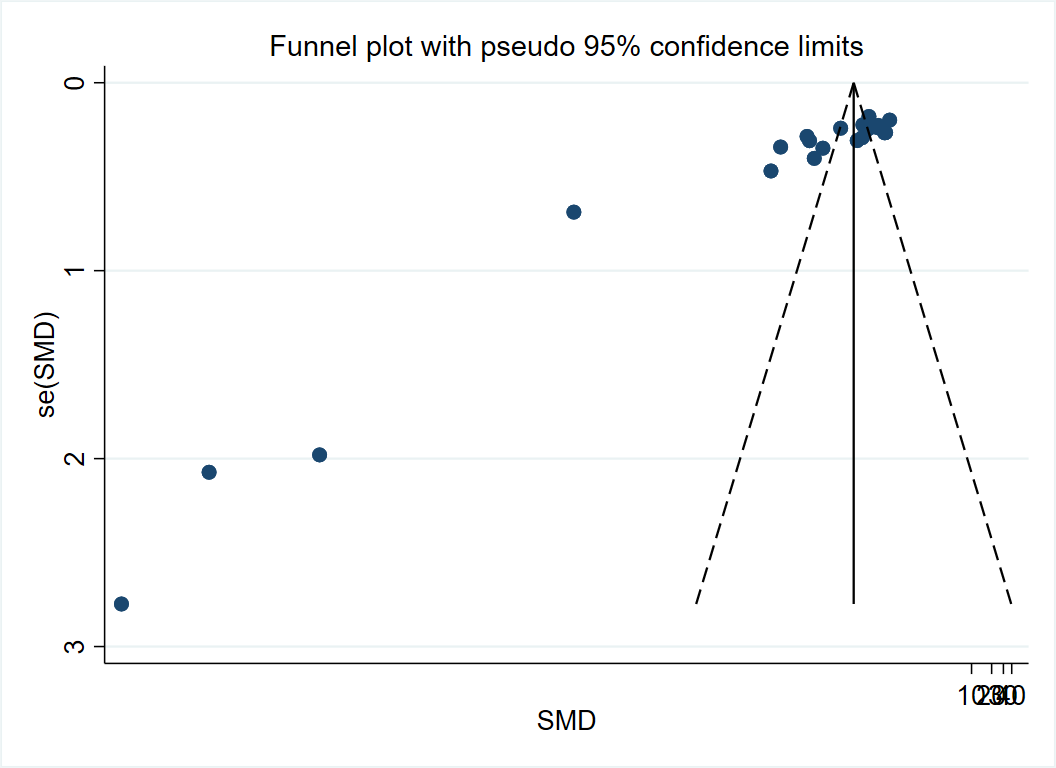


**Figure 3 First time out of bed after surgery**


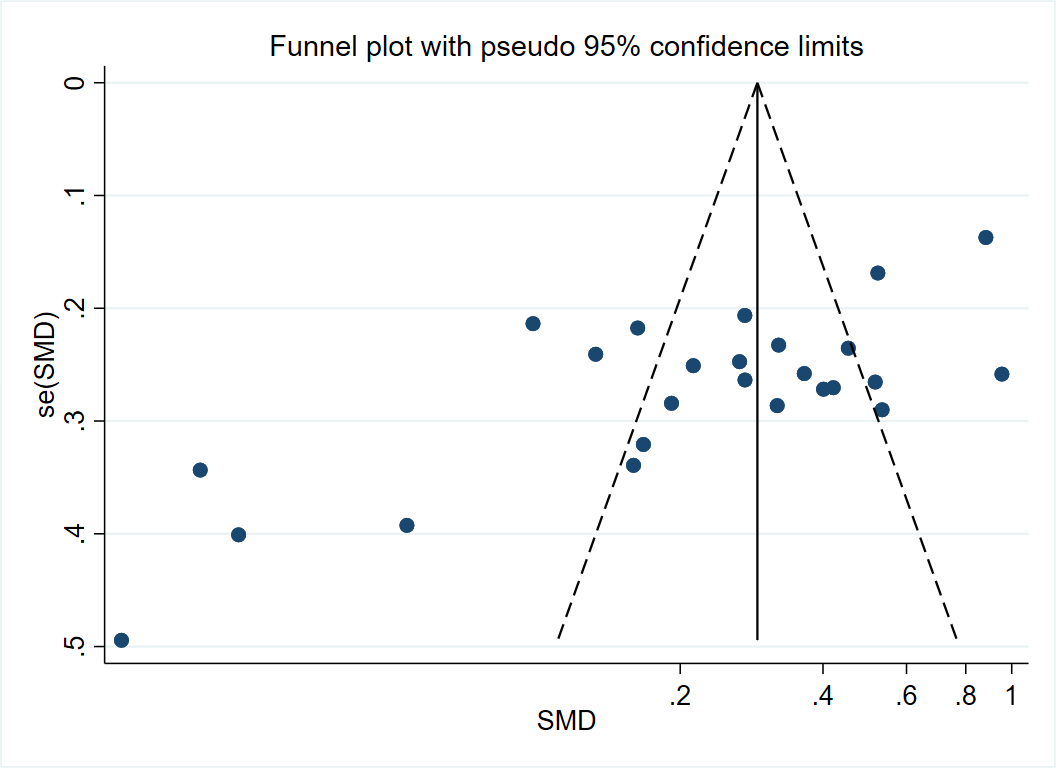


**Figure 4 Time of first postoperative anal exhaust**


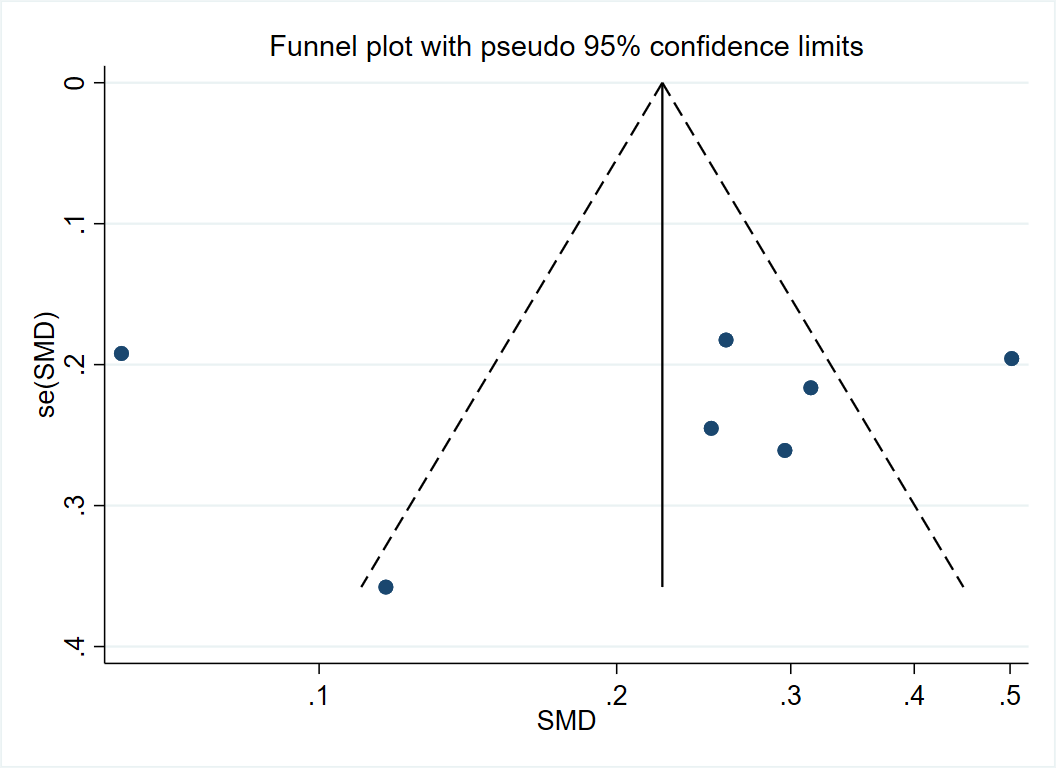


**Figure 5 Time of first bowel movement after surgery**


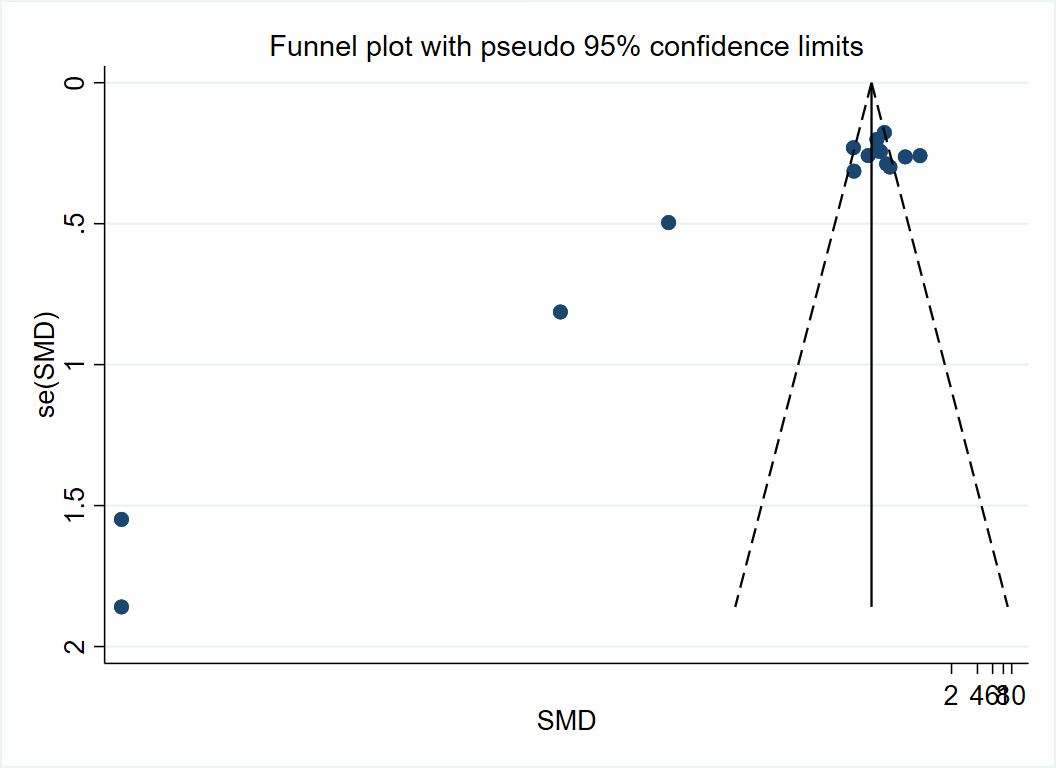


**Figure 6 Time of first postoperative food intake**


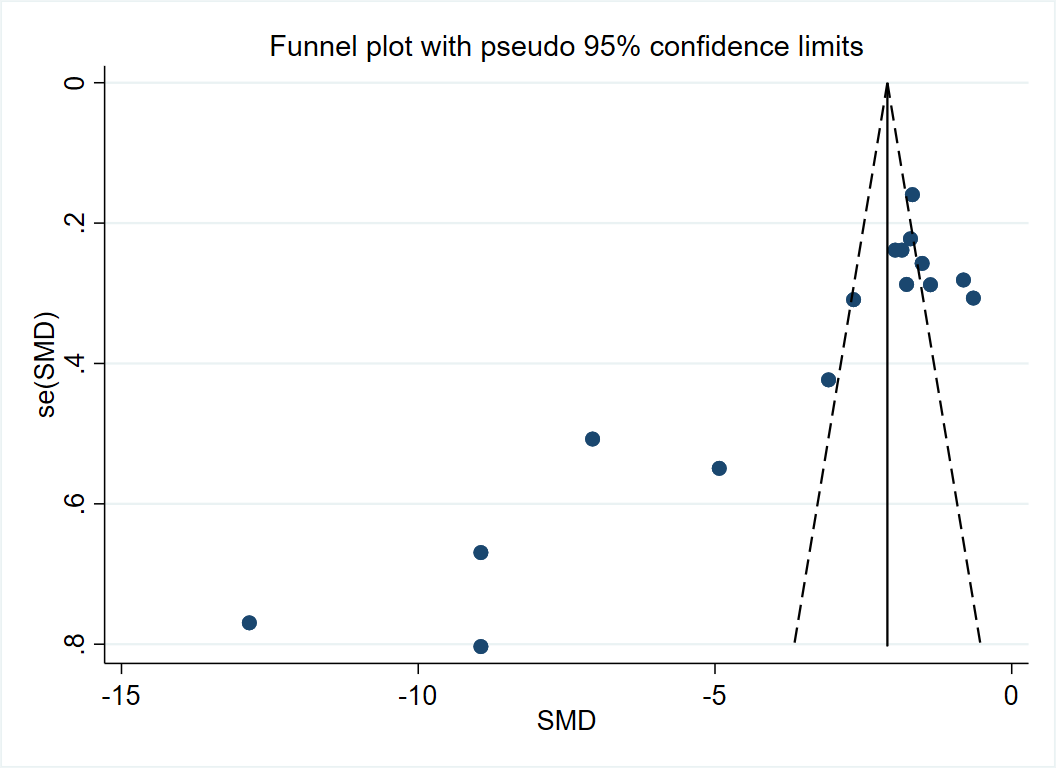


**Figure 7 Removal time of catheter**


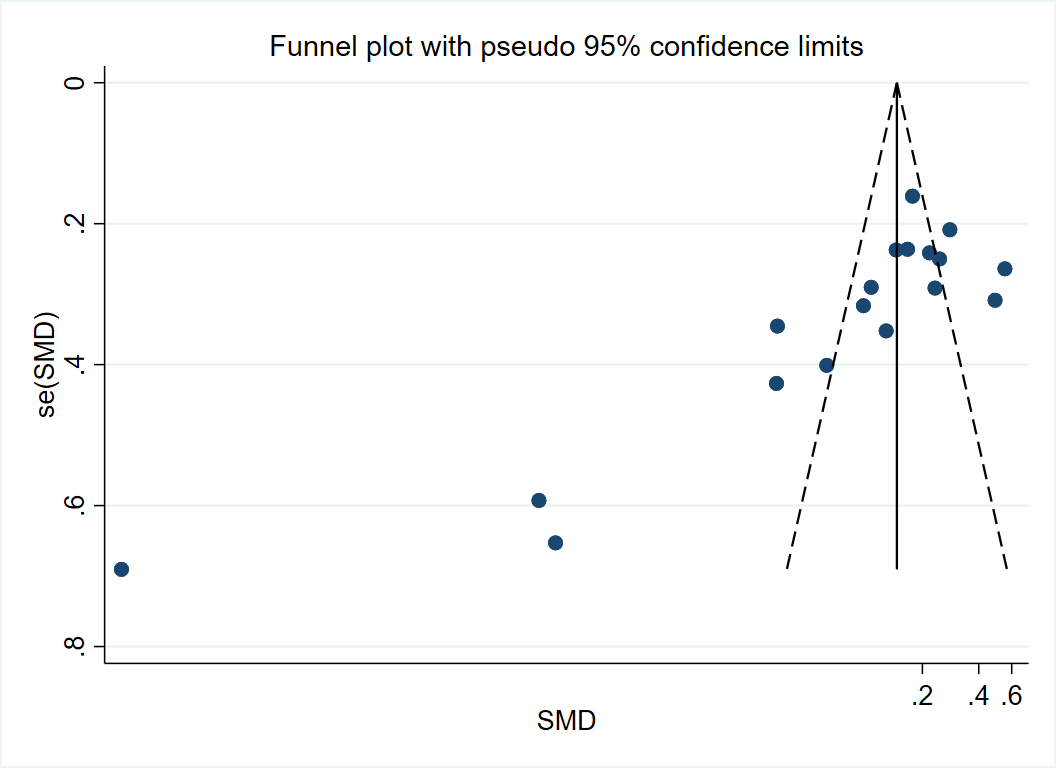


**Figure 8 Removal time of drainage tube**


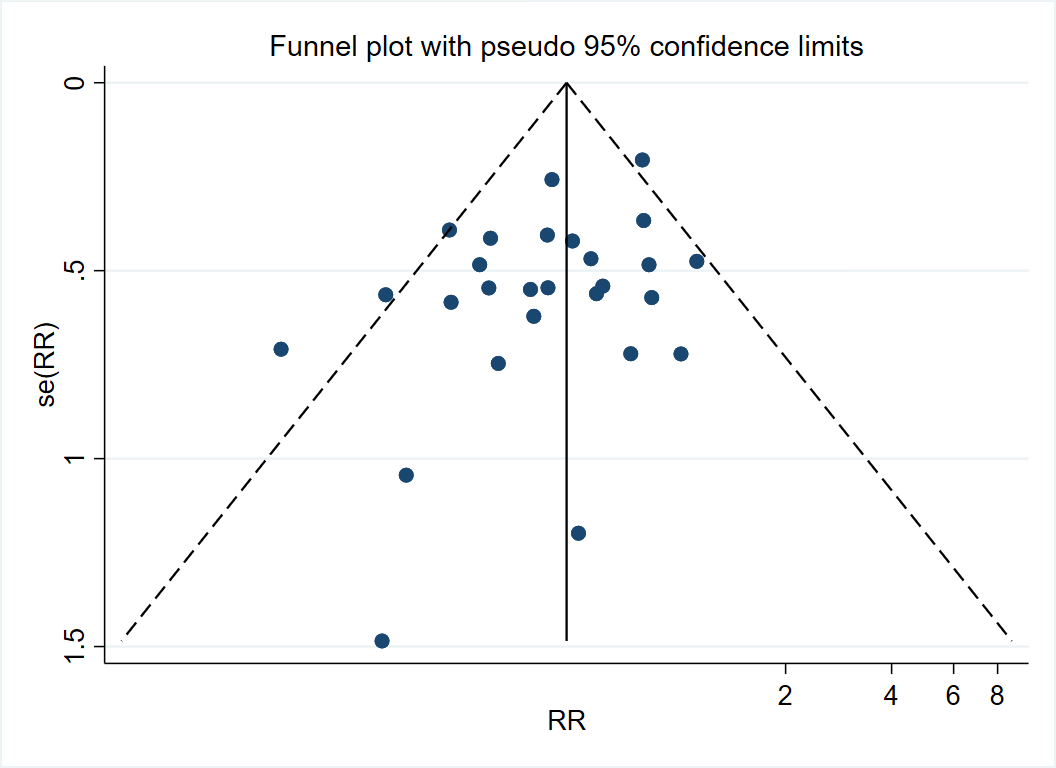


**Figure 9 Total postoperative complications**


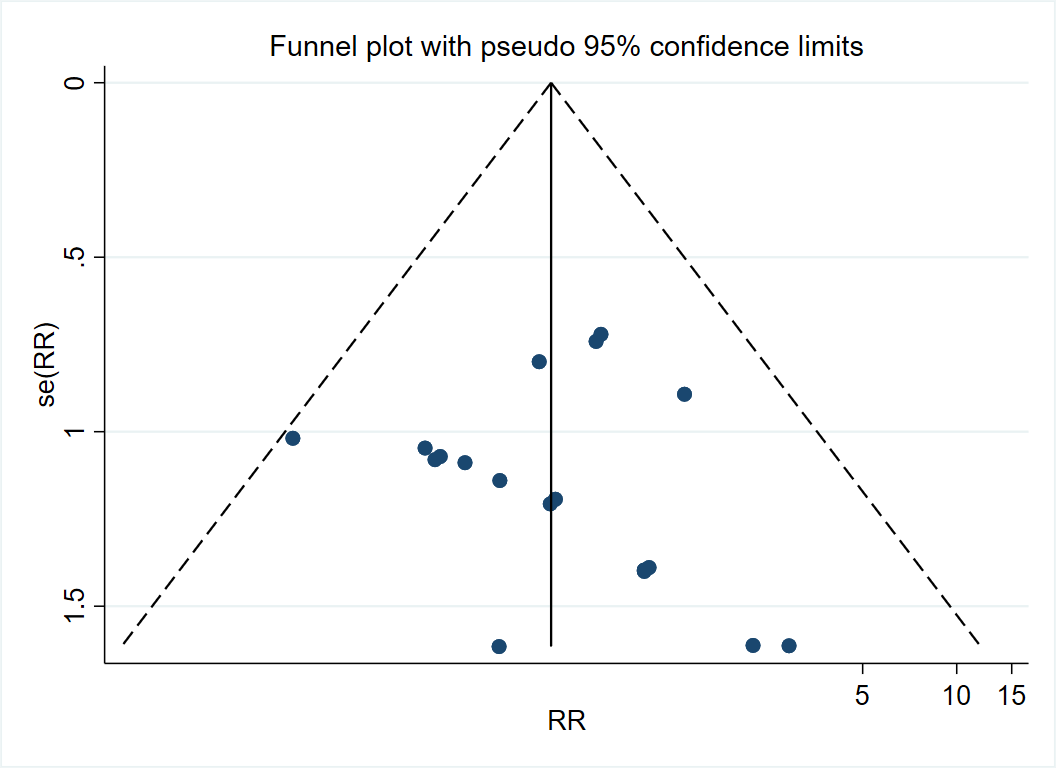


**Figure 10 Postoperative hemorrhage**


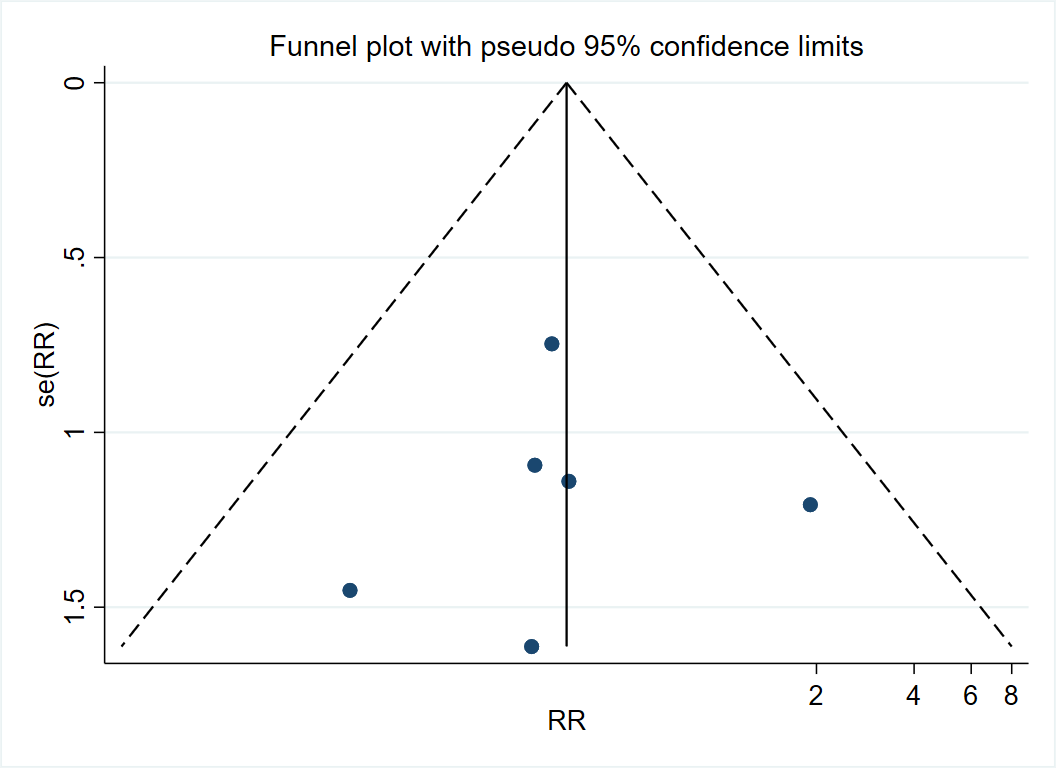


**Figure 11 Postoperative urine leakage**


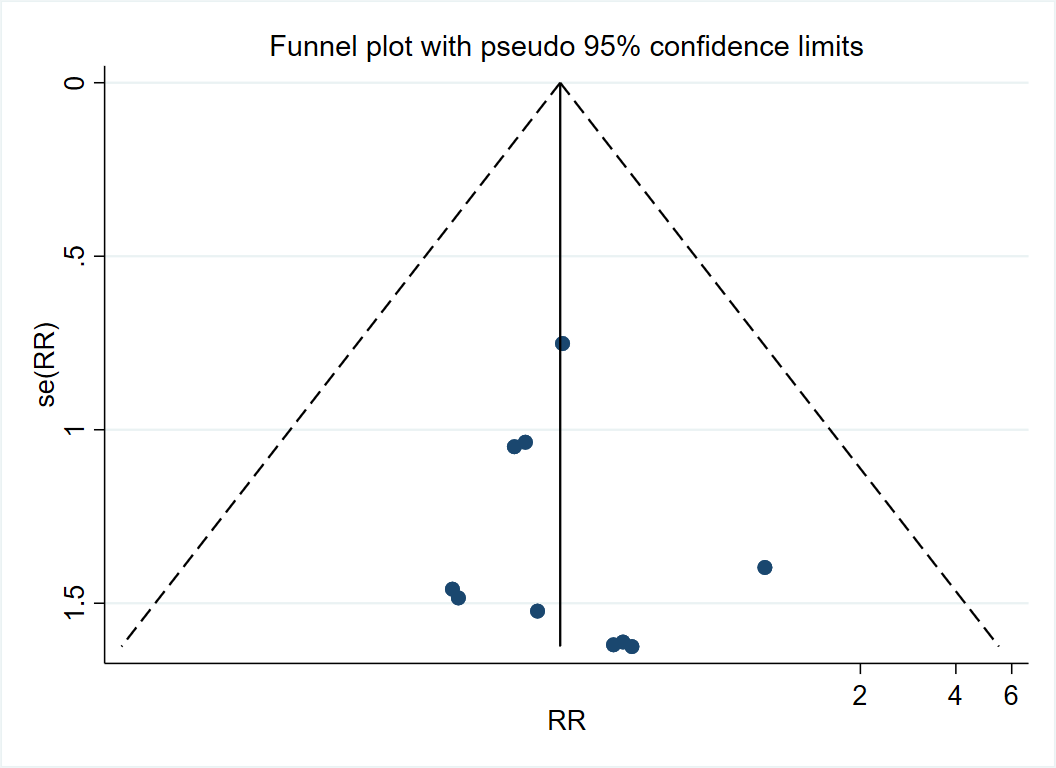


**Figure 12 Deep vein thrombosis**


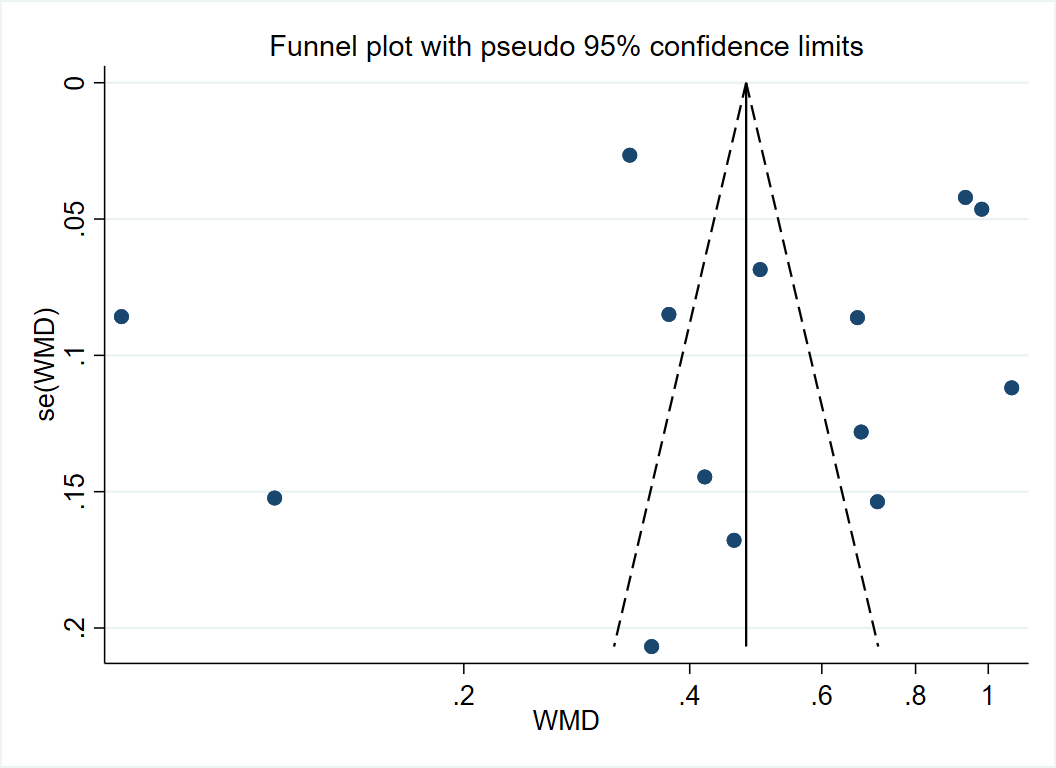


**Figure 13 Hospitalization costs.**
